# Single-cell polyadenylation site mapping reveals 3′ isoform choice variability

**DOI:** 10.15252/msb.20156198

**Published:** 2015-06-03

**Authors:** Lars Velten, Simon Anders, Aleksandra Pekowska, Aino I Järvelin, Wolfgang Huber, Vicent Pelechano, Lars M Steinmetz

**Affiliations:** 1 European Molecular Biology Laboratory, Genome Biology UnitHeidelberg, Germany; 2 Stanford Genome Technology CenterPalo Alto, CA, USA; 3 Department of Genetics, Stanford University School of MedicineStanford, CA, USA

**Keywords:** single-cell transcriptomics, alternative polyadenylation, transcript isoform, non-genetic heterogeneity, Bayesian inference

## Abstract

Cell-to-cell variability in gene expression is important for many processes in biology, including embryonic development and stem cell homeostasis. While heterogeneity of gene expression levels has been extensively studied, less attention has been paid to mRNA polyadenylation isoform choice. 3′ untranslated regions regulate mRNA fate, and their choice is tightly controlled during development, but how 3′ isoform usage varies within genetically and developmentally homogeneous cell populations has not been explored. Here, we perform genome-wide quantification of polyadenylation site usage in single mouse embryonic and neural stem cells using a novel single-cell transcriptomic method, BATSeq. By applying BATBayes, a statistical framework for analyzing single-cell isoform data, we find that while the developmental state of the cell globally determines isoform usage, single cells from the same state differ in the choice of isoforms. Notably this variation exceeds random selection with equal preference in all cells, a finding that was confirmed by RNA FISH data. Variability in 3′ isoform choice has potential implications on functional cell-to-cell heterogeneity as well as utility in resolving cell populations.

## Introduction

Cell-to-cell differences in gene expression are crucial to many processes in biology. Fluctuations in gene expression in single cells constitute symmetry-breaking cues in development (Ohnishi *et al*, 2014), affect the tolerance of cancer cells to chemotherapy (Spencer *et al*, 2009; Gupta *et al*, 2011), and allow microbes to thrive in alternating environments (Acar *et al*, 2008). On a molecular level, an important cause of variability in gene expression is the inherently noisy nature of biochemical reactions, where mRNA synthesis is an extreme case due to the low abundance of molecules involved in transcription (Raj & van Oudenaarden, 2008). In eukaryotes, the bursty nature of gene expression further increases the amount of noise. In a widely accepted model, eukaryotic genes of intermediate expression level typically switch between chromatin states that are prohibitive or permissive for transcription, leading to large differences in mRNA levels over time and across cells (Raser & O'Shea, 2004; Friedman *et al*, 2006; Sanchez & Golding, 2013). While it is well known that factors other than mRNA level, such as choice of mRNA untranslated regions (UTR), regulate protein expression and RNA function, it is not known to what extent UTR choice varies between single cells from genetically and developmentally homogeneous populations.

In mammalian cells, 3′ UTR sequence signatures on average exert a larger effect on protein levels than 5′ UTRs (Vogel *et al*, 2010). The 3′ UTR contains binding sites for miRNAs and RNA-binding proteins involved in control of mRNA translation, stability, and localization (Di Giammartino *et al*, 2011). 3′ UTR length is determined during transcription termination through alternative polyadenylation (APA), which affects about two-thirds of all human genes and thereby provides a mechanism to regulate gene expression independently of transcription level (Derti *et al*, 2012). 3′ UTRs globally lengthen during differentiation, but shorten during dedifferentiation and cancer formation (Ji & Tian, 2009; Ji *et al*, 2009; Mayr & Bartel, 2009). In many examples, it has been shown that APA alters transcript stability and translation rate, thereby affecting protein levels and cellular functions (Sandberg *et al*, 2008; Mayr & Bartel, 2009). While one recent genome-wide study in mouse embryonic fibroblasts has found that globally, such cases are relatively rare (Spies *et al*, 2013), two studies in yeast have shown that even single-nucleotide differences in 3′ UTR length can often lead to drastic changes in transcript stability and translation efficiency (Geisberg *et al*, 2014; Gupta *et al*, 2014). It is therefore possible that cell-to-cell variation in 3′ UTR choice contributes to phenotypic diversity.

Single-cell transcriptomics can provide important insights into cellular heterogeneity, and technology has rapidly advanced since it was first described (Tang *et al*, 2009). The use of cellular barcodes allows processing of multiple cells in single reaction tubes and has increased throughput to thousands of cells (Islam *et al*, 2011; Jaitin *et al*, 2014), while the use of microfluidics, especially in combination with molecular barcodes, has considerably decreased technical noise (Islam *et al*, 2013; Wu *et al*, 2014). Single-cell transcriptomics is therefore increasingly being used to chart the diversity of cell types within tissues (Jaitin *et al*, 2014; Treutlein *et al*, 2014) and during developmental transitions (Shalek *et al*, 2014), but investigation of heterogeneity within developmentally homogeneous cell populations has only recently started (Kumar *et al*, 2014). Following the availability of methods to determine the use of exons in single cells (Ramsköld *et al*, 2012), a recent study has investigated splice isoform heterogeneity in single dendritic cells (Shalek *et al*, 2013). Variability in 3′ UTR choice has so far, however, not been addressed, in part because of the lack of polyadenylation-site-specific single-cell transcriptomic protocols. Of equal importance, statistical methods have been developed to analyze gene expression heterogeneity over the background of technical noise in single-cell transcriptomic data (Brennecke *et al*, 2013; Grün *et al*, 2014), but approaches for analyzing isoform usage variability are currently lacking.

We characterized the extent of 3′ UTR choice variability in single cells on a genome-wide scale using BATSeq, a polyadenylation-site-specific single-cell transcriptomic protocol, and BATBayes, a computational framework for analyzing single-cell isoform data. Applied to three genetically and developmentally homogeneous stem cell populations, we find that individual cells differ in their preferences for polyadenylation (PA) sites. Random isoform choice with equal preference in all cells cannot explain the large observed variance. We show that especially in the case of low abundance transcripts, RNA 3′ isoform proportions are highly variable between cells, which may result in increased variations in post-transcriptional regulation. We further demonstrate that cell identity can be retrieved using information on 3′ end usage alone.

## Results

### BATSeq allows mapping and quantification of polyadenylation sites in single cells

To measure polyadenylation site usage in single cells, we combined the use of unique molecular identifiers (UMI) (Islam *et al*, 2013) with a highly accurate polyadenylation site mapping protocol (Pelechano *et al*, 2012) to develop a **BA**rcoded, **T**hree-Prime specific **Seq**uencing method (BATSeq).

**Figure 1:**
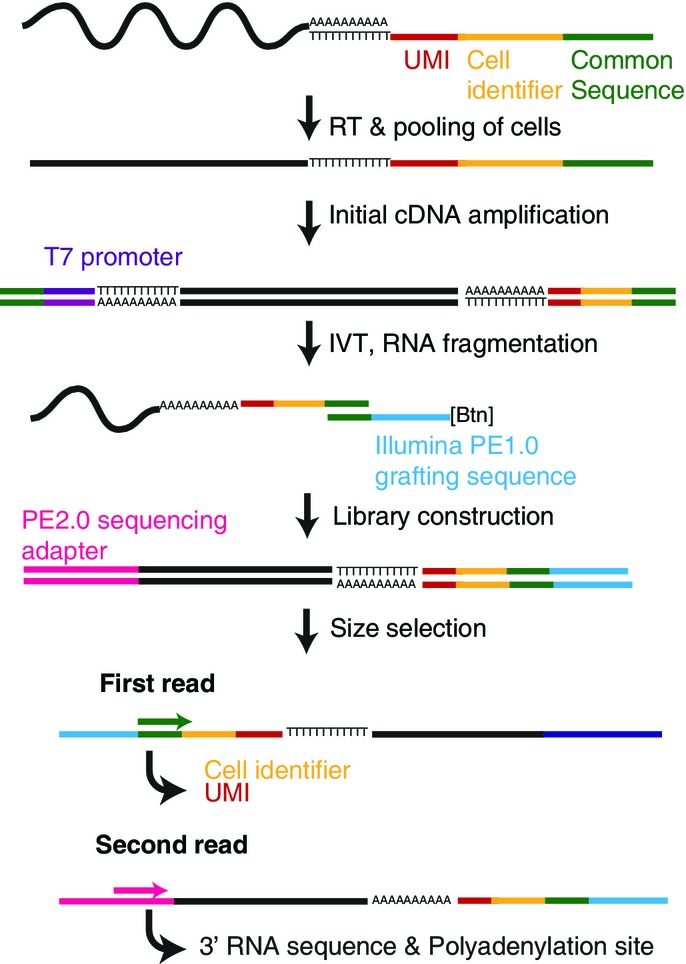
Scheme of the BATSeq protocol Reverse transcription was performed using an oligo-dT primer containing a unique molecular identifier (UMI), a cell identifier, and a common sequence. Following second-strand synthesis and limited PCR amplification, linearized RNA was produced by means of *in vitro* transcription (IVT). RNA was then captured at the very 3′ end using a biotinylated oligonucleotide, and libraries were produced on magnetic beads, followed by high-throughput sequencing.

In short, UMI and a cell barcode were incorporated during reverse transcription at the 3′ end of the mRNA poly-A tail (Fig1). cDNA amplification was performed with a limited number of PCR cycles following a published protocol (Sasagawa *et al*, 2013) and *in vitro* transcription. 3′ ends were captured using a biotinylated tag, followed by 3′ specific library construction (Pelechano *et al*, 2012). A short first sequencing read was used to obtain the UMI and cell barcode, whereas a longer (280 bases) second read was used to determine gene identity and polyadenylation site (Fig1 and Materials and Methods).

We applied BATSeq to 48 mouse embryonic stem cells maintained in medium with FCS and LIF (called ESC-FCS in the following), 48 ESCs maintained in medium containing LIF and the two selective inhibitors Chiron99021 and PD0325901 (called ESC-2i in the following) and 48 neural stem cells (NSC). To reduce large extrinsic fluctuations dependent on cell cycle state and cell growth (Snijder & Pelkmans, 2011), we FACS-sorted all cell populations by DNA content and size to include only small cells in G0/G1 (Appendix Fig S1). The libraries were sequenced on an Illumina MiSeq platform to a total depth of 42.3 million read pairs, 10.3 million of which passed computational filters as polyadenylation events (see Appendix Fig S2A and Materials and Methods for detail on read processing and filtering). We noted that sequencing existing libraries deeper did not substantially increase the number of observed barcodes, but that library complexity could be increased by repeating the final library amplification step directly from the magnetic beads (Appendix Fig S2B). We observed 869,000 unique transcript molecules (UMI-gene combinations) across the 144 sequenced cells. After discarding cells with fewer than 1,000 observed transcript molecules, 107 cells were included in the further analysis (Appendix Fig S2C).

To gauge the accuracy of BATSeq in mapping 3′ ends, we utilized spiked-in *in vitro* transcripts with known polyadenylation (PA) sites (ERCC RNA spike-ins). We observed that 95% of all identified polyadenylation events lay within 12 nucleotides of the annotated PA site (Appendix Fig S3); we therefore collapsed all observed putative polyadenylation events to the highest peak within 12 nt distance and excluded putative PA sites of very low observed frequency. Following this filtering strategy, all PA sites of the ERCC spike-ins were identified correctly, with no false positives.

**Figure 2:**
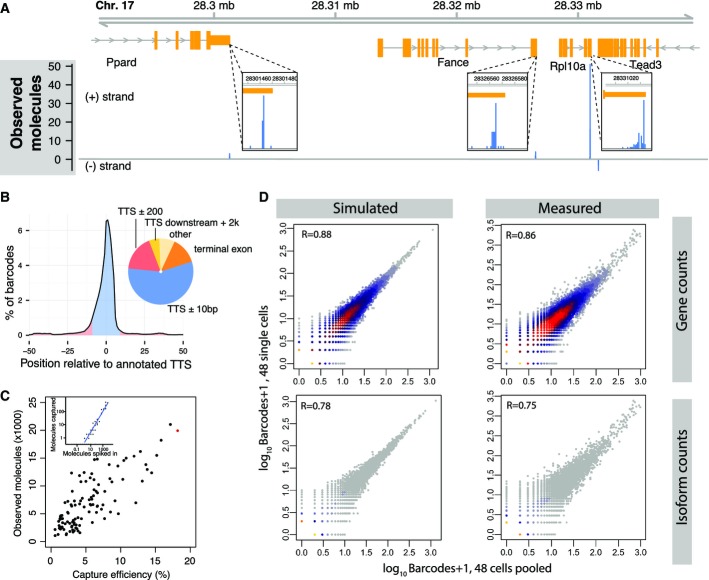
BATSeq provides quantitatively accurate polyadenylation site mapping in single cells

Of all putative polyadenylation events identified in the mouse genome, 56% lay within 10 nt of annotated polyadenylation sites; of the remainder, most events aligned to terminal exons or up to 2 kb downstream of annotated PA sites (Fig2A and B). Note that the current annotations cover many frequently used PA sites, but any specific tissue uses approximately 50% unannotated PA sites (Derti *et al*, 2012).

Mapping to a sample genomic region demonstrates that reads from BATSeq align to known 3′ ends of genes. Molecule counts from all cells were pooled, and alignment to a region of chromosome 17 is shown. Surrounding known polyadenylation sites, reads scatter typically by < 10 nucleotides (insets)

Global alignment statistics. Genes were aligned by transcript termination site (TTS). The distribution of barcodes mapped to different genomic features is shown.

Quantification of BATSeq capture efficiencies using *in vitro* transcript spike-ins. For each cell, the number of RNA spike-in molecules observed after sequencing (inset, *y*-axis) was regressed against the known number of molecules spiked into the reaction (inset, *x*-axis), thereby determining a molecular capture efficiency for each cell. The inset shows the spike-ins for cell ESC-2i_G1 (red in main plot) with its regression line; the main plot shows the results of these regressions for all cells. In reactions with higher capture efficiency, a higher number of cellular RNA molecules is observed (Pearson correlation 0.75).

Correlation between 48 ESC-FCS cells pooled (bulk experiment, *x*-axes) and 48 single cells (*in silico* sum of gene expression values, *y*-axes). The right panels show the measured correlations for gene counts (top) and isoform counts (bottom). The capture efficiencies of this experiment were somewhat lower (average of 0.04) than in our main dataset (Fig2C) due to less sequencing depth. To assess what correlations are expected from a noise model of binomial loss of RNA molecules (see Fig5A), we simulated two rounds of binomial subsampling from a distribution of “true” gene expression values, using measured capture efficiencies as success probabilities (left panels). Log gene expression values for the simulation were sampled from a mixture of two normal distributions, fitted to match, after subsampling, the measured distribution of the pooled cells. It is important to note that different strategies of selecting the distribution of “true” gene expression values had minimal effect on the simulated correlation coefficient. See Appendix Fig S2F for an alternative strategy.

In order to avoid biases introduced during cDNA amplification, we counted UMIs instead of reads, thereby increasing the average pairwise Pearson correlation between samples to *R* = 0.95 for the spiked-in *in vitro* transcripts and to *R* = 0.72 for the transcripts produced by the different cells (Appendix Fig S2D, average pairwise correlation of read counts: *R* = 0.92 and *R* = 0.6). The mean capture efficiency of BATSeq was estimated to be 5.4% by regressing the observed number of UMIs on the known concentration of the ERCC spike-ins (Fig2C). We observed a mean of 6,980 UMIs (transcript molecules) per cell, stemming from an average of 2,800 genes observed per cell. These benchmarks were similar to the values reported in a recently published single-cell transcriptomic method (Grün *et al*, 2014) which, unlike BATSeq, does not provide the ability to map polyadenylation sites. The frequency of UMI template switching was negligible (Appendix Fig S2E).

As an additional measure to gauge the quantitative precision of the BATSeq method, we generated libraries from a pool of 48 single ESC-FCS cells and compared them to the *in silico* average of 48 additional single cells generated on the same day. We observe a Pearson correlation of 0.86 for gene-level counts and 0.75 for isoform counts between these technical controls (Fig2D).

In the analyses presented below, we assume that technical noise in UMI-based methods is due to binomial sampling of a pool of RNA species with a known capture efficiency (Fig5A). To confirm that such a process accounts for all technical noise of BATSeq, we simulated bulk-vs.-single cell correlations based on that assumption (Fig2D, Appendix Fig S2F; see Figure legend for details on how simulations were performed). The obtained correlation of 0.88 for simulated gene-level counts and 0.78 for simulated isoform-level counts are very close to the measured values, and we therefore conclude that the technical noise of BATSeq is well described by binomial sampling. The small difference between experiment and simulation may be due to residual biological variance between two pools of 48 cells.

### BATSeq identifies known and novel genes with highly variable expression in stem cell models

**Figure 3:**
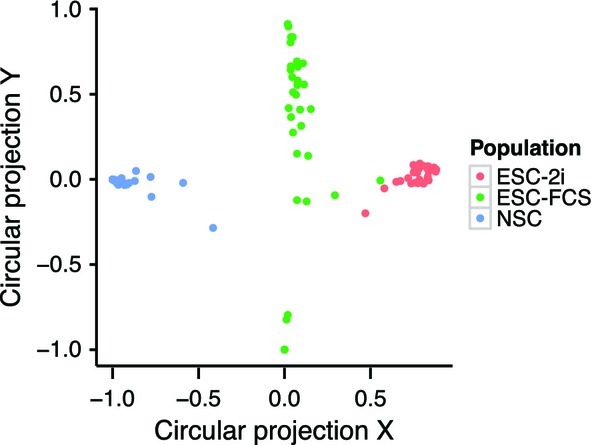
Single cells cluster by cell populations Molecular gene count data were used for the clustering. See Jaitin *et al* (2014) for the algorithm used for the projection.

To confirm that BATSeq can be used to derive single-cell gene expression, we first analyzed expression levels without taking isoform information into account. Expression of marker genes such as *Nanog*, *Sox2*, and *Nes* followed expected patterns in ESC-FCS, ESC-2i, and NSC populations (Fig EV1A), and cells readily clustered into the three populations (Fig3). We further confirmed that mean molecule counts measured in this study were well correlated with values published in two other studies, in which single-cell transcriptomics of embryonic stem cells was performed (Fig EV1B, Pearson correlation coefficients: Islam *et al*, 2013 – this study: 0.65, Grün *et al*, 2014 – this study: 0.72, Islam *et al*, 2013 – Grün *et al*, 2014: 0.73).

By applying a statistical method which tests whether observed gene expression variability exceeds what is expected from technical noise by at least a given margin (Brennecke *et al*, 2013), we identified genes with highly variable expression within each population. (Fig EV2, Table EV1). As expected, the transcription factors *Rex1* and *Nanog* appeared variably expressed in the ESC-FCS population (Chambers *et al*, 2007; Toyooka *et al*, 2008), but not in the ESC-2i population, for which a more homogeneous signaling state is expected (Wray *et al*, 2010). In ESC-FCS, the extent of expression variability for several other development-related genes was even higher; examples included the body-axis specifying signaling molecule *Lefty1* and the DNA methyltransferase regulator *Dnmt3l*.

Within the ESC-2i population, several genes displayed highly variable expression; examples include the transcription factor *Stella*, a regulator of the embryoid body/trophectoderm fate decision (Hayashi *et al*, 2008), and the mesenchymal stem cell marker *Sca-1*. The number of identified variable genes was smaller in ESC-2i than in ESC-FCS (Fig EV2D), in line with what was expected from the more homogeneous signaling state in this condition (Wray *et al*, 2010; Grün *et al*, 2014). NSCs again appeared to constitute a more heterogeneous population, with highly variably expressed genes enriched in the GO-term “cerebellum development” (*P* = 2.5 × 10^−4^, 6-fold enrichment compared to non-variably expressed genes).

We conclude that BATSeq can provide insight into gene expression of stem cell populations, and we confirm that some genes are variably expressed in ES cells maintained in 2i medium, a condition generally considered very homogeneous (Wray *et al*, 2010).

### Bayesian modeling reveals variability in isoform preference across single cells from homogeneous populations

**Figure 4:**
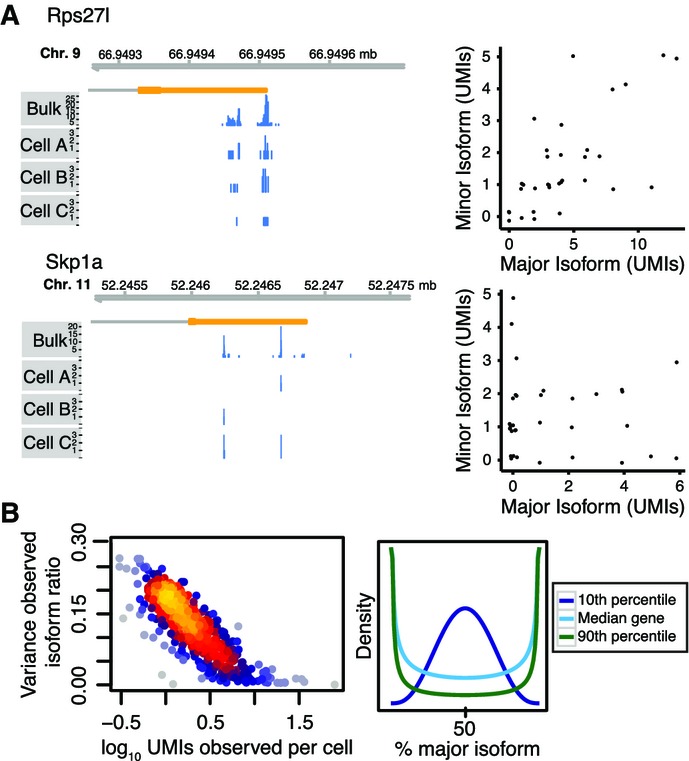
Raw isoform count data is noisy

We next sought to characterize variability in polyadenylation site usage within the relatively homogeneous stem cell populations. For some genes, such as the ribosomal protein *Rps27l*, observed ratios between major and minor 3′ isoform were similar in different cells within the ESC-2i population, that is, a higher expression of the gene was reflected by a proportional increase in the levels of both isoforms (Fig4A, upper panel). By contrast, for many other genes such as the ubiquitin ligase *Skp1a*, observed isoform ratios were highly variable within that population and expression levels of major and minor isoform did not appear correlated (Fig4A, lower panel).

Example genes. For some genes such as *Rps27l*, higher gene expression appears to result in a proportional increase in both isoforms; for other genes such as *Skp1a*, isoform usage appears not to be correlated at all. The left panels show coverage tracks for pooled data of all ESC-2i cells and three sample cells. The right panels show scatter plots summarizing data from all ESC-2i cells. Up to 0.1 UMIs xy-jitter was added to reduce overplotting.

Global trend. Overall, the variance in observed (raw) isoform ratios is lower in more highly expressed genes. The right panel illustrates hypothetical densities for the 10^th^, 50^th^ and 90^th^ percentile of observed variance and an assumed isoform ratio of 50:50.

To gain a comprehensive view, we focused on those 493 genes for which we observed at least two isoforms expressed at moderate to high levels each, corresponding to an average expression level of between 8 and 1,000 RNA molecules per cell and isoform (Table EV2). We rarely observed more than 2 isoforms per gene at that expression level, and we therefore restricted our analysis to the two most highly expressed isoforms of each gene. In the following, we focus on the ESC-2i population as an illustrative example because coverage for that population was highest (Appendix Fig S2C), but conclusions drawn for the other populations were identical and are included in the figures where appropriate.

**Figure 5:**
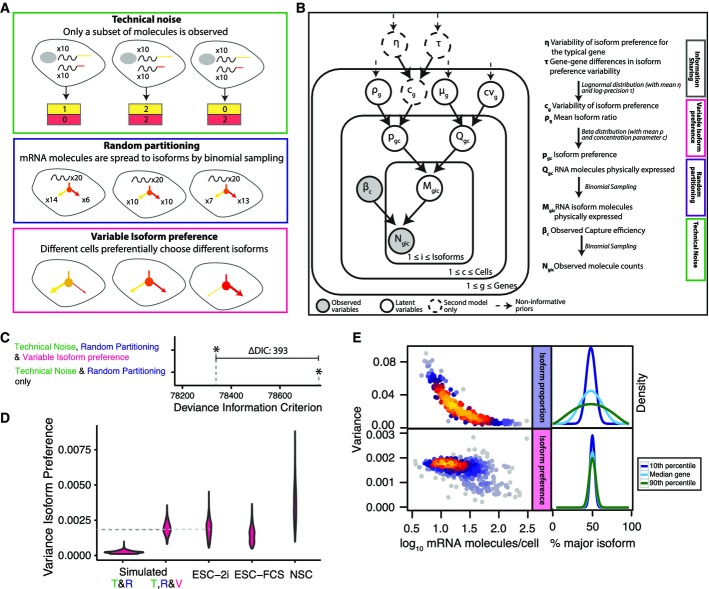
Isoform preference is different in different cells

At the level of the raw data, isoform ratios of highly expressed genes appeared less variable than isoform ratios of lowly expressed genes (Fig4B). Obvious causes of variability are technical noise in single-cell RNA sequencing (95% of RNA molecules are not observed) and random partitioning of the set of mRNA molecules to isoforms (Fig5A). Only if technical noise and random partitioning cannot explain the observed variability in isoform ratios, one has evidence of biological variability in the *preference* of single cells for different isoforms.

Three layers of noise can explain the observed variance in isoform ratios.

Directed acyclical graph of the BATBayes model. The number of RNA molecules per cell, Q_gc_, is drawn from a negative binomial distribution with parameters μ_g_ (the mean expression level of gene g), and cv_g_ (the coefficient of variation). All other parameters and distributions are explained in the figure. See also Appendix Supplementary Text and Supplementary Code EV1.

A model with variable isoform preference is to be preferred according to the Deviance Information Criterion.

Posterior of the variance in isoform preference is different from zero for real data. The posterior density of the variance in isoform preference was different from zero for real data, but concentrated close to zero for data simulated under the assumptions of a model of identical isoform preference in all cells (i.e., all variability due to technical noise & random partitioning only). The model provides a quantitatively correct estimate of the variance in a dataset simulated under the assumption of a specific variance in isoform preference (dashed light grey line indicates the value assumed during simulation, dark grey line indicates the inferred value).

Global distribution of inferred variance of isoform ratios. For lowly expressed genes, considerable variance in isoform proportion exists solely due to the effect of binomial partitioning of RNAs to isoforms. Isoform preference is less variable. The level of variance is similar across genes and independent of gene expression level. The right panel illustrates hypothetical densities for the 10^th^, 50^th^ and 90^th^ percentile of variance and an assumed isoform ratio of 50:50.

We developed and compared two Bayesian statistical models (“BATBayes”) to dissect the relative contribution of technical noise, random partitioning, and putative variability in isoform preference (see Fig5B, Appendix Supplementary Text and Supplementary Code EV1 for an explicit mathematical presentation of our model). In these models, we describe PA site choice as a stochastic process, as follows: Whenever a cell produces a transcript molecule for a gene with several PA sites, the PA site used for the new molecule will be chosen at random, with each of the available PA sites having a certain probability of being chosen. We refer to this vector of probabilities as the cell's *isoform preferences* for the given gene. We then ask whether all cells within a population have the same isoform preferences (first model) or whether isoform preferences vary from cell to cell (second model). Here, it is important to distinguish the isoform preferences from the *isoform proportions*. By the latter, we mean the proportions of a gene's isoforms among the transcript molecules that were actually present in the cell at the time of lysis. Even in the first model (same isoform preferences in all cells), the isoform proportions will differ from cell to cell, in the same way as two runs of each ten times flipping a coin may result in two different counts of heads. We refer to the latter effect as the *random partitioning* of the set of the transcript molecules present in a cell into polyadenylation isoforms (Fig5A, blue box). If now, in our coin analogy, two differently biased coins are used in the two runs, the observed counts will tend to differ in a more extreme way; and in a similar way, we will see stronger variability if the isoform preferences vary across cells (Fig5A, red box). Note that not only isoform preferences but also isoform proportions cannot be directly observed: Due to the limited capture efficiency of single-cell sequencing, a molecule is seen in the sequencing libraries only with a certain probability, giving rise to further variation, which we describe as technical noise (Fig5A, green box).

In our first model, only technical noise and random partitioning contribute to cell–cell variability in polyadenylation site usage, whereas the second model allows for different cells to have different isoform preferences. The second model shares information across genes to infer the variability in isoform preference for the “typical” gene (Fig5B), but also infers gene-wise estimates of isoform preference variability, which we discuss further below.

We found clear evidence for variability in isoform preference, that is in favor of the second model, based on the following analyses: We first used the deviance information criterion (DIC; Spiegelhalter *et al*, 2002), which compares models based on goodness of fit and expected degree of overfitting and found that the DIC evaluated in favor of the second model (Fig5C, ΔDIC: 393). We then fitted the second model to the data and found that the variance in isoform preference (for the typical gene) was estimated to be different from zero for all stem cell populations under study (Fig5D, see also Appendix Fig S4 for details on model fitting using Monte Carlo Markov chains). In contrast, when we simulated a dataset with no variability in isoform preference, we found an estimate that was close to zero (Fig5D, and Appendix Fig S5A for details on the simulated dataset). When we simulated a dataset with a known variance in isoform preference, the inferred posterior mean of the variance parameter deviated from the value used for the simulation by < 1% (Fig5D). We further used simulations to verify that the inferences made do not depend on accurate estimates of the capture efficiency of BATSeq (Appendix Fig S5B and C). The conclusions drawn from the model even hold if capture efficiencies differ for different isoforms (Appendix Fig S5D). We finally compared whether the variability in isoform preference differs in the different cell types under study, and we found that it was quantitatively similar in all cell types under study.

We therefore conclude that BATBayes constitutes a useful framework for disentangling different sources of cell–cell variability in isoform choice, and we show that polyadenylation site usage is variable across single cells. To confirm this finding by an independent, more frequentist statistical approach, we compared the variance of the observed isoform ratios for each gene to the expected variance obtained by simulating the first model 1,000 times for each gene (Appendix Fig S6A). We found a significant enrichment of genes whose observed variance exceeded the variance predicted by the first model (*P* = 4.6 × 10^−8^, Binomial test).

For the entire set of genes analyzed, lowly expressed genes had more variable isoform proportions. This is because both technical noise and also noise from random partitioning (Fig5E, upper panel) increase in strength for low transcript counts. Importantly, the BATBayes model does not only infer a global parameter for isoform choice variability, but does provide gene-by-gene estimates; however, we found only evidence for relatively minor gene–gene difference in isoform choice variability (Fig5E, lower panel and Appendix Fig S6B and C). Larger isoform-specific single-cell sequencing datasets may in future help to more clearly disentangle gene-wise differences in isoform choice variability.

### Isoform choice variability is also evident from smFISH

**Figure 6:**
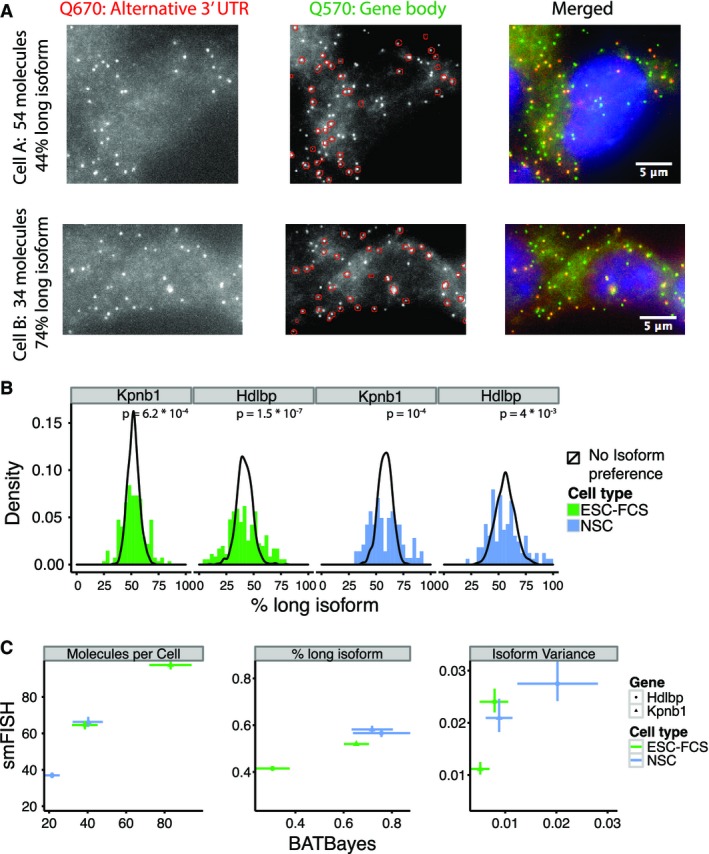
smFISH validates isoform choice variability

To investigate single-cell isoform usage with an independent experimental method, we performed RNA-isoform-specific smFISH in ESC-FCS and NS cells (Waks *et al*, 2011). Two genes (*Kpnb1* and *Hdlbp*) were selected based on the following criteria: (i) length difference between alternative isoforms over 500 nt; (ii) expression of both isoforms at approximately equal amounts; (iii) a total expression level of between 30 and 100 molecules per cell to facilitate spot counting. We designed Q570-labeled probes specific to the common sequence and Q670-labeled probes specific to the optional region of the 3′ UTR. Specificity of the probes was confirmed by checking for co-localization of signal from the alternative 3′ UTR with signal from the common sequence (Fig6A; Appendix Fig S7). In all experiments, we observed examples of cells using mostly either the long or the short isoform (Fig6A; Appendix Fig S7). We then quantified the number of spots (Materials and Methods) and found that the distribution of isoform ratios across cells was significantly broader than what would be expected if only random partitioning, but no variable isoform preference, were to determine isoform ratios (Fig6B). Tendencies of mean number of molecules per cell, average isoform ratio, and also the variance of isoform proportions matched between the BATBayes prediction and smFISH (Fig6C). smFISH therefore validates the qualitative and quantitative predictions of the BATBayes model.

Raw smFISH data for a sample gene, *Hdlbp*. Shown are one NS cell with a low percentage of mRNA molecules using the long isoform (top row) and one cell with a high percentage of mRNA molecules using the long isoform (bottom row). Left column: Red channel containing probes specific to the alternative 3′ UTR. Central column: Green channel with probes specific to the gene body. Dots identified in the red channel are superimposed in red to demonstrate that each red dot colocalized with a green dot. Right column: Both channels merged.

Distribution of isoform ratios observed by smFISH, contrasted to distributions expected assuming no variability in isoform choice. *P*-values shown are from a Kolmogorov–Smirnov test comparing simulated and measured distributions.

Quantitative comparison of different parameters inferred by the two methods. Error bars denote 66% confidence intervals.

In single-molecule FISH, bright nuclear spots are interpreted as active sites of transcription that contain several recently produced mRNA molecules which have not yet diffused off their site of synthesis (Raj *et al*, 2006; Waks *et al*, 2011). For the *Kpnb1* gene in ES cells, we found that such sites are dominated by a single isoform (Fig EV3). This observation provides a first hint at the mechanism behind isoform choice variability: Once set, an active polyadenylation site appears to remain active for several cycles of transcription.

### Coordinated changes in 3′ UTR length dominate isoform preference in mixed populations

**Figure 7:**
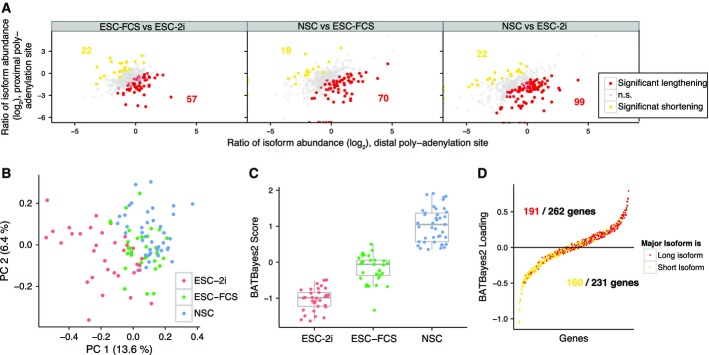
Clustering of single cells based on 3′ isoform usage

Large coordinated changes in 3′ UTR length are frequently observed across cell populations and during development (Sandberg *et al*, 2008; Ji *et al*, 2009). By pooling data from single cells of the three different stem cell populations, we found evidence for the use of longer 3′ UTR isoforms in neural stem cells, and, interestingly, in ESC-FCS compared to ESCs maintained in 2i medium (Fig7A). To investigate whether isoform preferences can be used to identify single cells independently of gene expression, we fitted the BATBayes model to the pool of all 107 cells included in this study. Cell types were roughly separated based on the estimates of single-cell isoform preference, but ESC-FCS cells did not form a distinct cluster (Fig7B). We improved the clustering by extending BATBayes to include a component of correlated changes in 3′ isoform preference (Fig EV4A, Appendix Supplementary Text and Supplementary Code EV2). When we fitted the extended model (BATBayes2) to all 107 cells, cell populations were separated completely (Fig7C). The observed clustering appeared to be predominantly due to coordinate lengthening from ESC-2i to NSC of the 3′ UTRs of almost all genes under study (Fig7D). The BATBayes2 algorithm was designed such that only isoform choice, but not total gene expression levels, influences the clustering. Indeed, simulated datasets confirm that clustering by BATBayes2 is not affected by gene expression levels (Fig EV4B); further, the cell types also separated well if using only genes expressed at similar levels (average expression fold changes of < 2, Fig EV4C). The 3′ UTR usage pattern therefore contains all the information required to identify cell types. We further note that the 3′-based clustering algorithm developed here separates cell types comparable to circular *a posteriori* projection, a state-of-the art algorithm based on total gene expression levels (Jaitin *et al*, 2014; see Fig3).

Molecule count data from pooled cells reveals a trend toward isoform lengthening from ESC-2i to ESC-FCS and NSC. Data from all cells of each population were pooled. Red dots indicate genes for which the longer 3′ isoform was significantly upregulated in the population under investigation; yellow dots correspond to significant upregulation of the shorter 3′ isoform. Significance was determined by Fisher's test (*P *<* *0.05), see also Hoque *et al* (2013).

Clustering of cells based on isoform preference. BATBayes estimates for isoform preference were subjected to principal component analysis.

BATBayes2 can be used to effectively cluster cells from different populations based exclusively on 3′ isoform use. Posterior means of the inferred scores are shown; each dot corresponds to a cell. For details on the model applied, see Appendix Supplementary Text, Fig EV4A and Supplementary Code EV2

BATBayes2 reveals coordinate lengthening of 3′ isoforms. Posterior means of the inferred loadings are shown; each dot corresponds to a gene. Mild jitter in *y* direction was added to reduce overplotting.

We then asked whether coordinate changes in 3′ UTR choice also govern isoform variability within homogeneous populations. Importantly, the BATBayes2 model is not restricted to coordinated 3′ UTR length changes, but designed to discover any correlations in 3′ UTR choice across single cells. When we fitted the extended model to the individual cell populations, we found no evidence for the presence of a correlated component. Simulations showed that relatively strong correlations across at least half of the genes studied are required for such an effect to be noticeable given the current data (Fig EV4D). While we therefore cannot exclude the presence of some correlated variation, for example, due to fluctuations in miRNA expression, we conclude that effects affecting multiple genes in a coordinate fashion do not dominate isoform choice variability.

## Discussion

The major finding from our study is that even in homogeneous populations, cells differ in their preferences for 3′ RNA isoform choice. This variability is beyond what can statistically be explained by either technical noise or random partitioning of RNA molecules to isoforms.

To investigate cell-to-cell heterogeneity in polyadenylation site usage on a genome-wide scale, we developed BATSeq, the first method for PA-site quantification in single cells. BATSeq accurately identifies known PA sites and its quantitative accuracy is comparable to recently published protocols that do not include 3′ isoform information (Grün *et al*, 2014). In future, the use of microfluidics has the potential to increase quantitative accuracy further (Islam *et al*, 2013; Wu *et al*, 2014). Like others, we found that the use of molecular barcodes in single-cell transcriptomics is useful, not only because of reduced technical noise, but also because the availability of molecular counts facilitates quantitative statistical modeling of the biological processes that generate the observed data (Grün *et al*, 2014; Jaitin *et al*, 2014).

Polyadenylation requires the recruitment and binding of the 3′ end processing machinery to the nascent pre-mRNA, where the efficiency of recruitment depends on the affinity of the polyadenylation signal to the processing machinery (Gil & Proudfoot, 1987). The 3′ end processing machinery is expressed in relatively limiting amounts (Chuvpilo *et al*, 1999), and it may therefore be expected that due to stochastic binding of PA factors to nascent mRNAs, polyadenylation would for each nascent transcript occur randomly at either site. While such a mechanism alone could create considerable cell-to-cell heterogeneity in 3′ isoform usage ratios, our results demonstrate the presence of an additional source of variability.

For mRNA levels, it has been shown that variability exceeds molecular noise, both due to mechanisms acting at the level of individual genes and due to mechanisms that collectively affect multiple genes (Elowitz *et al*, 2002; Raser & O'Shea, 2004). In the case of mRNA isoforms, a possible mechanism affecting multiple genes would, for example, be variations in the expression of core polyadenylation machinery components or miRNAs. However, we found no evidence for coordinated variation in polyadenylation site choice (Fig EV4D). Mechanisms that affect genes individually are therefore likely to contribute to the variability in isoform preference. In the case of mRNA levels, transcriptional bursting greatly increases gene expression noise (Raj & van Oudenaarden, 2008), and analogous mechanisms might affect isoform preference. For one gene, we observed that active sites of transcription are dominated by a single 3′ isoform. Once chosen, an active polyadenylation site might remain active for several cycles of transcription; however, this proposed mechanism warrants further investigation.

In bulk data, it has previously been shown that PA site choice is affected by nucleosome density and certain chromatin marks (Spies *et al*, 2009; Khaladkar *et al*, 2011). Variability in chromatin states between individual cells may therefore affect polyadenylation site activity and be a mechanism that confers isoform choice variability. While beyond the scope of this study, dynamic measurements of isoform usage in single cells, for example, based on live RNA labeling (Hocine *et al*, 2013), could provide further insights into the molecular mechanisms involved.

In the case of mRNA levels, expression variability serves to diversify cellular phenotypes (Acar *et al*, 2008; MacArthur & Lemischka, 2013). Our results show that especially for genes of moderate expression, polyadenylation isoform ratios are highly variable across cells solely because of random partitioning of mRNAs to isoforms. In cases where isoforms differ in stability (Spies *et al*, 2013), it is therefore conceivable that random isoform choice translates to differences in protein expression, similar to examples known from bulk studies, where altered isoform use of single genes can even affect cell proliferation (Mayr & Bartel, 2009). Stochastic variations in 3′ isoform usage may therefore be an additional cause of phenotypic cell–cell heterogeneity, at least in the case of some genes.

Between the three stem cell populations investigated, 3′ isoform usage changes in a coordinated fashion for many genes, with more pluripotent populations (ESC-2i, ESC-FCS) expressing shorter 3′ UTRs. This finding is in line with previous work that found 3′ UTRs to gradually lengthen during development, especially neuronal development (Ji *et al*, 2009), and to shorten during dedifferentiation to iPS cells (Ji & Tian, 2009). Interestingly, we found that single cells from different populations of stem cells can be clearly distinguished by 3′ UTR usage, independently of gene expression levels. This demonstrates that 3′ UTR usage is under tight control during developmental changes or alterations in signaling environment (ESC-2i vs. ESC-FCS). While earlier work demonstrated that such isoform shortenings have clear phenotypic consequences at least in some cases (Mayr & Bartel, 2009), the transcriptome-wide consequences of global length changes are unclear (Spies *et al*, 2013; Gruber *et al*, 2014). It would also be conceivable that UTR length changes are just a consequence of epigenomic alteration between ESC-2i, ESC-FCS, and NSC, which remains to be further characterized. From a practical perspective, single-cell RNA isoform preference clearly contains information useful for distinguishing between cell types and may therefore increase resolution in single-cell transcriptomic studies aimed at charting tissue heterogeneity.

Some recent work has investigated cell–cell variability in RNA splicing. While a study using single-molecule FISH on two genes found that variability in splice isoform ratio exceeds the variability expected from random partitioning to a relatively modest level, quantitatively similar to the level we observed for polyadenylation isoforms (Waks *et al*, 2011), a single-cell transcriptomic study reported much more widespread bimodality in splice isoform usage (Shalek *et al*, 2013). BATBayes could help to reconcile these findings by accounting for both technical noise of single-cell transcriptomics and the probabilistic distribution of RNA molecules to isoforms.

In conclusion, the results presented here demonstrate that variability in 3′ isoform use is a layer of transcriptomic heterogeneity that has previously been overlooked, despite its potential implications on regulation of transcript isoform choice and its utility in separating cell populations.

## Materials and Methods

### Stem cell culture

We used 46C mouse embryonic stem cells (Sox1-GFP ES cells) (Ying *et al*, 2003), originally derived from the E14tg2a cell line. Cells were grown at 37°C in a 5% (v/v) CO_2_ incubator on gelatin-coated (0.1% v/v) dishes. For the ESC-2i samples, serum-free ES cell culture medium (2i/LIF) was prepared by supplementing the 50% Dulbecco's modified Eagle's minimal essential medium, 50% F12 (DMEM/F12, Invitrogen) medium with N2 and B27 (Gibco), BSA (Gibco), HEPES (final concentration 4.5 mM), 0.1 mM beta-mercaptoethanol and with PD0325901 (1 μM), CHIR99021 (3 μM), and LIF (10 ng/ml, produced in-house). For the ESC-FCS samples, the ES cells were grown in Glasgow modified Eagle's medium (GMEM, Invitrogen), supplemented with 10% (v/v) fetal bovine serum (FBS) (Sigma), LIF (10 ng/ml, produced in-house), 1 mM beta-mercaptoethanol, non-essential amino acids (Gibco), and sodium pyruvate (Gibco). Accutase (Sigma) was used for cell dissociation. Cells were passaged every second day at a seeding density of 3 million cells per 10-cm petri dish. Medium was exchanged daily.

### Differentiation of ES cells to neural cells and culture of NS cells

To initiate monolayer differentiation into NS cells (Ying & Smith, 2003; Ying *et al*, 2003; Pollard *et al*, 2008), ES cells were plated at a density of 2 million cells per gelatin-coated 10-cm petri dish in 50% Dulbecco's modified Eagle's minimal essential medium, 50% F12 (DMEM/F12, Invitrogen) medium supplemented with N2 and B27 (Gibco), BSA (Gibco), non-essential amino acids (Gibco), glucose (final concentration 0.03 M), HEPES (final concentration 4.5 mM), and 0.1 mM beta-mercaptoethanol (differentiation medium). Medium was exchanged after 24 and 48 h, and the cultures were grown for additional 72 h. Cells were then gently dissociated using Accutase (Sigma), the GFP^+^ cell fraction (corresponding to ca. 70% of cells) was sorted by flow cytometry and seeded into a laminin (Sigma)-coated 75-cm^2^ flask (final density of laminin: 10 μg/cm^2^ of culture surface, coating time: minimum 4 h at 37°C). Subsequently, cells were grown in differentiation medium supplemented with 10 ng/ml in-house-prepared recombinant murine EGF and bFGF until loss of GFP expression and uniform up-regulation of Nestin expression was observed. Cells were passaged at 80% confluence, and medium was exchanged daily.

### FACS sorting for single-cell transcriptomics

Cells were detached as described above, taken up in culture medium, and stained with Hoechst 34580 (Life Technologies) at a dilution of 1:10 for 15 min. Small cells with 1N DNA content were then sorted by gating for Hoechst fluorescence and Forward/Side-Scatter (Appendix Fig S1).

### cDNA synthesis and amplification

For initial cDNA amplification, a modified version of the QUARTZ-Seq protocol (Sasagawa *et al*, 2013) was used. During all steps described in the following, reactions were kept on ice. Individual cells were sorted in 0.6 μl of *single-cell lysis buffer* (for a list of all buffers used in BATSeq, see Appendix Table S1) containing ERCC spike-ins at a final dilution of 1:4,000,000. Primers were annealed by addition of 0.8 μl *priming buffer* followed by 90 s of incubation at 70°C and 15 s of incubation at 35°C. Reverse transcription was performed by addition of 0.8 μl *barcoding RT buffer* and 5 min of incubation at 35°C, 20 min at 45°C, and 10 min at 70°C. The RT primers contained barcodes for early multiplexing (see Appendix Table S2 for a list of primers used); however, to avoid bead purification steps that potentially compromise capture efficiency, only cells from 4 neighboring wells were pooled at that stage. For digestion of unbound primer, 4 μl of *ExoI buffer* was added and primer digestion was performed by 30 min of incubation at 37°C, followed by 20 min of inactivation at 80°C. Restricted poly-A tailing was performed by addition of 10 μl *polyA tailing buffer* and incubation at 37°C for 50 s, followed by enzyme inactivation for 10 min at 65°C. Second-strand synthesis was performed by addition of 35 μl *PCR Mix I* and incubation at 98°C for 130 s, 40°C for 1 min, and 68°C for 5 min. The primer used for second-strand synthesis contained a T7 promoter that was later used to linearize the PCR product. Suppression PCR was performed by addition of 50 μl *PCR Mix II* and 14 cycles of denaturing (98°C, 10 s), annealing (65°C, 15 s), and synthesis (68°C, 5 min), followed by a final synthesis step (68°C, 5 min).

PCR product was purified by addition of 0.6× HighPrep™ PCR beads (MAGBIO) and elution to 10 μl elution buffer. The volume ratio of magnetic beads to PCR was chosen to select against short products, that is, by-products that formed from poly-A tailing of remaining RT primer (see Tang *et al*, 2009; Sasagawa *et al*, 2013). Following bead purification, neighboring wells were pooled. Each well then contained the amplified cDNA from 8 cells.

### *In vitro* transcription

The PCR product was further amplified and prepared for 3′ end capture by *in vitro* transcription (IVT). Therefore, 10 μl *IVT mix* (Appendix Table S1) were added to 20 μl of purified cDNA and incubated at 37°C for 14 h, followed by enzyme inactivation for 10 min at 65°C. cDNA was digested using the Turbo DNA-free kit (Life Technologies) according to the manufacturer's instructions. The amplified RNA was purified by addition of 1.3× the reaction volume of HighPrep™ PCR beads (MAGBIO) and eluted into 10 μl EB. Neighboring wells were pooled so that each well finally contained the reaction product from 24 uniquely barcoded cells in 30 μl volume.

### Library construction for poly-A site mapping

To map poly-A sites, a published protocol (Pelechano *et al*, 2012) was modified. Amplified RNA was fragmented by addition of 7.5 μl *fragmentation buffer* (Appendix Table S1) and incubation at 80°C for 7 min. Cleanup was performed using 1.8× HighPrep™ PCR beads and elution into 10 μl EB. Reverse transcription was performed using a capture primer complementary to the 5′ region of the primer used in the first step of cDNA synthesis and amplification. First, 1.7 μl 1.57 M trehalose, 0.5 μl 1 μM biotinylated BATSeq capture primer (Appendix Table S2), and 1 μl 10 mM dNTP mix (NEB) were added. Samples were then incubated at 65°C for 5 min to disrupt secondary structure, and subsequently, 7.3 μl *library RT buffer* was added and samples were incubated at 42°C for 50 min, followed by enzyme inactivation at 72°C for 15 min. Cleanup was performed using 1.5× HighPrep™ PCR beads and elution into 39.5 μl EB. Second strands were synthesized by addition of 10.5 μl of DNA polymerase I and RNaseH-containing *second-strand buffer*, followed by incubation at 16°C for 2.5 h. To remove primers and short products, cleanup was performed using 1× HighPrep PCR beads™ and eluted in 20 μl EB.

Libraries were then constructed on magnetic beads. 20 μl of Dynabeads M-280 Streptavidin (Invitrogen) were washed two times with 200 μl *B&W buffer* and resuspended in 20 μl 2× B&W buffer. Purified cDNA was then added to the beads and incubated for 15 min at room temperature. Dynabeads were washed twice using B&W buffer, once using EB and resuspended in 21 μl EB. End repair was performed by addition of 2.5 μl end repair buffer and 1.25 μl end repair enzyme mix (NEBNext DNA Sample Prep Master Mix Set 1, NEB) and incubation at 20°C for 30 min. The beads were washed as before, and again resuspended in 21 μl EB. A-tailing was performed by addition of 2.5 μl 10× NEBuffer 2 (NEB) supplemented with 0.2 mM dATP and 1.5 μl Klenow fragment (3′-5′- exo^−^, NEB), followed by 30 min of incubation at 37°C. Beads were washed as before, and resuspended in 10.2 μl EB. To each batch of 24 pooled cells, a sequencing adaptor containing a specific “batch” barcode was annealed by addition of 0.8 μl of the corresponding P7_T1_Mpx linker at a concentration of 0.5 μM (Appendix Table S1), 1.5 μl 10× T4 DNA ligase buffer (NEB), and 2.5 μl T4 DNA ligase (2,000 U/ml, NEB). Samples were incubated for 1.5 h at 16°C, and beads were washed 4 times in B&W buffer, once in EB, and resuspended in 24 μl EB. Enrichment PCR was performed by the addition of 0.5 μl of 10 μM PE2.0 primer, 0.5 μl 10 μM PE1.BATSeq primer, and 25 μl 2× Phusion HF master mix (NEB); 30 s of incubation at 98°C; and 20 cycles of denaturing (98°C, 10 s), annealing (68°C, 10 s), and synthesis (72°C, 10 s), followed by a final extension step (72°C, 5 min). Supernatant containing PCR product was taken off the Dynabeads and purified using 1.8× HighPrep™ PCR beads. Product was loaded on an E-Gel 2% SizeSelect, and fragments of a length of 200–350 bases were selected.

### cDNA sequencing

cDNA sequencing was performed on an Illumina MiSeq platform using a custom sequencing primer for the first read, TATAGAATTCGCGGCCGCTCGCGAT. The first read was stopped after 20 cycles (sufficient to obtain cell & molecular barcode), and the second read was continued for 280 cycles. To obtain deeper sequencing, enrichment PCR was repeated two times from stored beads. In total, four MiSeq runs were performed (see Appendix Fig S2A).

### Read pre-processing, alignment, and filtering

For processing the sequencing data, we made use of the HTSeq Python package (Anders *et al*, 2014), and custom scripts written in Perl and Python. The first seven bases of the second reads of the fragments were trimmed off and used to demultiplex the sequencing reads into batches; for each batch, the first six bases of the first read were trimmed off and used to demultiplex the reads into reads stemming from individual cells (see also Fig1). The next eight bases of the first read contain the molecular barcode, which was trimmed off and stored. All further processing was exclusively on the second read. Terminal As were trimmed off, and only reads with at least 10 terminal As were retained. By using GSNAP, version 2012-01-11 (Wu & Nacu, 2010), these reads were aligned to the Mus musculus genome, assembly GRCm38 (downloaded from ENSEMBL, version 38.73), to which we had appended the sequence of the ERCC spike-ins. Alignments were then filtered to exclude non-uniquely aligned reads, alignments of low quality (below a mapping quality of 30), short reads (below 20 bp length), and reads containing more than 80% A. To avoid signal stemming from false priming, reads were further filtered to exclude all reads that stem from regions of the genome containing more than 80% A in a window of 15 bases downstream of the mapped 3′ end, or more than 65% A in a window of 50 bp downstream of the mapped 3′ end. Reads mapping to the mitochondrial genome or rRNAs were removed from all further analyses, as these RNA species are polyadenylated during degradation by processes that are independent of APA (Nagaike *et al*, 2005; Slomovic *et al*, 2010).

### Molecule counting and identification of polyadenylation sites

We first counted the number of reads for each unique combination of 3′ alignment position and molecular barcode. We then determined, for each 3′ alignment position, whether it maps to a known gene or downstream of a known gene within a window of 20 kb; if so, the read was annotated as stemming from said gene. The relatively large (20 kb) window was used to account for the recent finding that a considerable amount of transcription that was previously thought to stem from intergenic transcription actually stems from 3′ UTRs, especially in neural tissues (Miura *et al*, 2013); however, in our dataset, long 3′ UTRs are rather an exception (< 2.5% of molecules mapped more than 2 kb downstream of an annotated TTS, see also Fig2B). If no gene could be identified, reads were counted as antisense (if they were antisense to a known gene) or intergenic. We thus obtained a table listing gene identifier, alignment position, molecular barcode, and read count. We split this table by gene identifier and used a published method (Qu *et al*, 2009) to merge, for each gene, all molecular barcodes that were not sufficiently distinct (Hamming distance of one or less). We observed virtually no instances where the 3′ ends of reads with identical molecular barcodes were not in immediate proximity (< 20 bp distance); any such instances were discarded. We thus obtained a table listing gene identifier, polyadenylation site position, barcode, and read count.

To define reference polyadenylation sites, the data from all cells were merged and the number of unique molecular barcodes mapping to each genomic position was counted. In the case of ERCC spike-ins, we observed that estimated polyadenylation site positions scattered within a window of 12 bp surrounding the expected site (Appendix Fig S3). We therefore sorted, for the merged data of all cells, polyadenylation site positions by barcode count and, starting at the bottom of the list, checked whether we could identify a polyadenylation site with a higher barcode count within a 12-base pair window. If so, the site was eliminated and its barcode count was added to the identified polyadenylation site. We thus, over the population of all cells, obtained a table of estimated polyadenylation sites, the corresponding barcode count and assigned gene identifier. For each individual cell, we then assigned each alignment position to the position of the closest polyadenylation site identified over the entire population. We thus obtained tables of gene identifiers, estimated polyadenylation sites and barcode counts, with identified polyadenylation sites being compatible across different cells. We used these tables for all further analysis.

### Isoform-specific smFISH

For the *Hdlbp* and *Kpnb1* genes, 48 Q670-labeled probes specific to the alternative 3′ UTR and 48 Q570-labeled probes specific to the common sequence were designed using the Stellaris probe designer (Biosearch Technologies, CA). For cell fixation and hybridization of probes, we followed the protocol provided by Biosearch Technologies. Z-stacks of 6 images at a z-distance of 0.4 μm were taken of at least 80 cells per gene using a Zeiss CellObserver inverted fluorescence microscope.

Following maximum intensity projection, cells were segmented manually; spots were identified using a Laplacian-of-Gaussian filter and a threshold that was manually set to optimize the agreement between computational and visual identification of spots. Noise was removed using a morphological opening, and nearby spots were separated using a morphological watershed.

### Bayesian modeling

Models were fit to the data using JAGS (Plummer, 2003). For detailed information and model formulation, please refer to Appendix Supplementary Text. Model source code is supplied as Supplementary Codes EV1 and EV2.

### Data visualization

Data were visualized using the R programming language and the packages ggplot2 (Wickham, 2009), LSD, NeatMap, and Gviz.

### Data access

The data reported in this paper have been deposited in GEO under accession number GSE60768.

## Supporting Information

Appendix

Expanded View Figures PDF

Table EV1

Table EV2

Supplementary Code EV1

Supplementary Code EV2

Review Process File
