# Supplementary material for: Single-cell polyadenylation site mapping reveals 3′ isoform choice variability
Source: Mol Syst Biol. 2015 Jun 3;11(6):812. doi: 10.15252/msb.20156198 (PMC4501847; doi:10.15252/msb.20156198)
Supplement: Supplementary file 1 [file msb0011-0812-sd1.pdf]

Appendix to *Single cell polyadenylation site mapping reveals 3' isoform choice variability*

Lars Velten, Simon Anders, Aleksandra Pekowska, Aino I Järvelin, Wolfgang Huber, Vicent Pelechano and Lars M Steinmetz

Contents

1 Appendix Supplementary Text: Bayesian Modeling 1
- 1.1 Selection of genes for modeling 1
- 1.2 Formulation of the BATBayes model 1
- 1.3 Model fitting and comparison 5
- 1.4 Simulation of control data sets 5
- 1.5 BATBayes2: Clustering of single cells based on polyadenylation site usage 6

2 Appendix Figures S1-7 9

3 Appendix Table S1: List of buffers used for BATSeq 16

4 Appendix Table S2: List of primers used for BATSeq 18

1 Appendix Supplementary Text: Bayesian Modeling

1.1 Selection of genes for modeling

Due to the relatively low capture efficiency of single-cell transcriptomics, we observe only a single strongly expressed 3' isoform for most genes. We therefore restrict the following analysis to those 493 genes for which we observe at least two isoforms at an average of at least 0.2 barcodes per cell each, which corresponds to approximately 8 RNA molecules per isoform in each cell based on the estimates of the model (see Table EV2 for an overview of all genes included). For the majority of genes retained, only two isoforms are expressed at that level; we therefore restrict the following analysis to the two most abundant isoforms for each gene.

1.2 Formulation of the BATBayes model

To separate the observed variance into technical noise, variance induced by random partitioning of mRNAs to isoforms and potential contributions

by variance in isoform preference (Figure 5a), we developed two Bayesian models. The null model ("first model") is that isoform preference is the same in all cells, whereas the alternative model ("second model") allows isoform preference to vary (see Figure 5b for a visual depiction of both models). For each combination of gene g, polyadenylation isoform i, and cell c, we observe N_{gic} molecular barcodes. To account for *technical noise*, we model N_{gic} as a sample of the number of RNA molecules M_{gic} expressed in the cell

$$N_{gic} \sim \mathrm{Binom}(M_{gic}, \beta_c) \tag{1}$$

where β_c is the capture efficiency observed in cell c. β_c is determined by regressing the number of observed ERCC spike in molecules against the number of molecules put into the reaction (Figure 2c, inset). For an overview of all quantities used in BATBayes, see inline table 1.

N_{gic}	Number of RNA molecules of gene g and Isoform i observed in cell c
β_c	Capture efficiency in cell c
M_{gic}	Number of RNA molecules of gene g and Isoform i physically expressed in cell c
Q_{gc}	Number of total RNA molecules of gene g and physically expressed in cell c
μ_g	Average total expression of gene g
τ	Rate parameter for expression of gene g
p_{gc}	Probability of choosing Isoform 1 of gene g in an individual cell c
ρ_g	Average Probability of choosing Isoform 1 of gene g
c_g	Concentration of p_{gc} around ρ_g
η	Mean of c_g
τ	Log-Precision of c_g

Table 1: Quantities used in BATBayes. First subsection contains observed variables, second subsection contains latent variables.

For both models, we assume that upon expression of an RNA molecule, the isoform is chosen randomly with a gene- (and possibly cell-) specific probability p_{gc}. If a total of Q_{gc} RNA molecules are expressed in a given cell, isoform 1 is chosen M_{g1c} times according to binomial partitioning of RNA molecules to two isoforms:

$$M_{g1c} \sim \mathrm{Binom}(Q_{gc}, p_{gc}) \tag{2}$$

and

$$M_{g2c} = Q_{gc} - M_{g1c} \tag{3}$$

It is evident that under these assumptions, the observed isoform ratios can be highly variable even if p_{gc} is equal in all cells merely due to technical

noise and binomial partitioning. For the first model, we therefore assume equal isoform preference in all cells:

$$p_{gc} = \rho_g \tag{4}$$

In the second model, we assume that p_{gc} is variable across cells. We model this using the conjugate prior of the binomial distribution in (2), the Beta distribution:

$$p_{gc} \sim \mathrm{Beta}(a_g, b_g) \tag{5}$$

which we parametrize by its mean

$$\rho_g = \frac{a_g}{a_g + b_g} \tag{6}$$

and the so-called concentration parameter, a quantity related to the variance:

$$c_g = \sqrt{a_g^2 + b_g^2} \tag{7}$$

We chose this parametrization because c_g scales independently of the mean, whereas the variance is dependent on the mean (Appendix Figure S4a). We further note that this parametrization allows the model to easily be extended to more than two isoforms by using a Dirichlet distribution as the generalized form of the Beta distribution. For ρ, we assume a flat $0, 1$ prior:

$$\rho_{gc} \sim \mathrm{Unif}(0, 1) \tag{8}$$

c_g is unknown, but of primary interest to our model as it describes the cell-cell variability in isoform choice. For $c \to \infty$, the Beta distribution becomes narrowly peaked and the model degenerates to the first model. In a simpler version of the second model ("second model A"), we assume that c_g is equal for all genes:

$$c_g = 1/\eta \tag{9}$$

i.e. that all genes display equal variation in isoform preference. We parameterize by $1/\eta$ based on the consideration that at large values of the concentration parameter, even large changes have little effect on the resulting Beta distribution; a Beta distribution with concentration parameter 100 is almost identical to a Beta distribution with concentration parameter 200, but radically different from a Beta distribution with concentration parameter 0. We then place an exponential hyperprior on η:

$$\eta \sim \exp(1) \tag{10}$$

We note that the prior variance of η was 1, and the posterior variance of was 0.00007 or smaller, indicating that η was well defined by the data and that the exact choice of prior does not influence the conclusions drawn by the modeling.

We then allowed c_g to vary across genes ("second model B"). Initially, we fitted second model A to all genes independently. We observed similar values for c_g for most genes, but some outliers with very low concentration parameters (Appendix Figure S6c, x-axis). When we investigated these outliers, we found that they were mostly genes with very low expression level, i.e. typically only one barcode per cell observed. As beta distributions with extremely high variance (all density at 0 and 1) do not contain more prior uncertainty than extremely low variance beta distributions (all density at ρ), Bayesian parameter estimation greatly favored high-variance distributions in these cases. We therefore share information across genes and allow c_g only to deviate from the typical variance in isoform preference if the data justifies it by using an empirically estimated lognormal prior on c_g:

$$\log(c_g) \sim \mathcal{N}\left(\log\left(\frac{1}{\eta}\right) - \frac{1}{2\tau}, \sqrt{1/\tau}\right) \tag{11}$$

We use the lognormal distribution because as described before, at small concentration parameters, smaller absolute changes in c translate to larger changes in the Beta distribution than at large concentration parameters. The distribution was parameterized by its mean η and a precision parameter τ, upon which we place a conjugate hyperprior:

$$\tau \sim \exp(0.1) \tag{12}$$

The posteriors of τ are only shifted against the prior by 30% (Appendix Figure S6b). Based on the DIC and simulations, we use second model B as final model ("BATBayes"), but we note that the prior on τ influences the spread of final estimates of gene-wise variances displayed in the lower panel of figure 5e. The use of a rather conservative exponential prior with rate 0.1 avoids an overestimation of gene-wise differences that is not backed up by the data. To model the number of RNA molecules for any gene, we assume a negative binomial distribution, based on the observation that RNA molecule counts are typically more disperse than a Poisson distribution [5]:

$$Q_{gc} \sim \text{NBinom}(\mu_g, q_g) \tag{13}$$

parametrized by its expected value and rate parameters, for which we assume flat priors:

$$\mu_g \sim \text{Unif}(0.01, 10000) \tag{14}$$

$$q_g \sim \text{Unif}(0, 1) \tag{15}$$

The BATBayes source code is given in Code EV1.

1.3 Model fitting and comparison

We fit the model to the data (molecule count tables N_{gic} and capture efficiencies β_c) by using JAGS, a program for Bayesian parameter estimation using Monte Carlo Markov Chains [3]. Starting parameters for mean gene expression and isoform ratio can readily be estimated from the data; for all other parameters, we initialize at values drawn randomly from the prior distributions. For each combination of model and data considered in the main text, we ran at least 3 chains for at least 300,000 iterations each. We recorded values of ρ, η, τ, c, μ and q at each 100th iteration to avoid autocorrelation. We further monitored the variance of the isoform ratio $N_{g1\cdot}/(N_{g1\cdot} + N_{g2\cdot})$, which were used for creating parts of figure 5e. We further recorded the posterior means of all latent variable.

We use the CODA package of the programming language R to analyze MCMC chain output [4]. We discard a burn-in of up to 100,000 iterations and make sure that chains converge by using Gelman and Rubin's diagnostic [1], which was consistently below 1.1, as well as by manual inspection of diagnostic plots (see Appendix Figure S4b-d for representative examples). The deviance information criterion was computed using the JAGS DIC module. The DIC is defined as

$$\text{DIC} = \widehat{D_{avg}}(y) + p_D \tag{16}$$

Where $\widehat{D_{avg}}(y)$ is the mean deviance of data y across samples from a MCM chain, and p_D is the number of free parameters [6]. Akaike's Informaiton Criterion (AIC) and Schwartz' BIC are not applicable in the context of hierarchical Bayesian models because parameters can be closely constrained by their priors. p_D is estimated from the difference between the average deviance and the deviance at the posterior mean of the parameters:

$$p_D = \widehat{D_{avg}}(y) - D_{\hat{\theta}}(y) \tag{17}$$

1.4 Simulation of control data sets

To ascertain that the model provides correct estimates of variability in isoform preference, we simulated several control data sets and investigate the behavior of the model fit. We first simulate data under the assumptions of the second model B (isoform preference is variable across different cells, and this variance is different in different genes) by drawing random values for N, M, Q, p and c from the distributions specified in equations 1-3, 5 and 9. As parameters for ρ, η, τ, μ and q, we assume the posterior means of the model fit to the ESC-2i data. For β, we assume the measured values. The parameter estimates obtained from fitting the second model B to the simulated data deviate from the true values by consistently less than 30%; the estimate of the important hyperparameter η deviates from the real value by less than 1% (Figure 5d).

We then simulated data under the assumptions of the first model (isoform preference is equal in all cells) by drawing random values for N, M and Q from equations 1-4, again assuming the posterior means of the model fit to the ESC-2i data as parameters. The variance of isoform preference obtained from fitting the second model B to this simulated data set is correctly estimated to be close to 0, approximately 10 times lower than the value estimated for the real data (Figure 5d).

Besides the observed data N, the model depends on the capture efficiencies β, which were estimated based on the use of spiked-in in vitro transcripts. To make sure that the conclusions drawn from the model are insensitive to experimental inaccuracies in determining β, we simulated data under the assumption of the first model (no variability in isoform preference) and one of the following scenarios. In the first scenario, we assume that the true value β' is really lower than the experimental estimate β by a fixed factor

$$\beta'_c = \varphi \cdot \beta_c \tag{18}$$

In the second scenario, we assume that β' is different for different genes

$$\beta'_{cg} = \mathcal{S}(\text{Logit}(\beta_c) + e_g) \tag{19}$$

were $\mathcal{S}$ is the sigmoid function and

$$e_g \sim \mathcal{N}(0, \varphi) \tag{20}$$

In the third scenario, we assume that β' is different for different genes and isoforms

$$\beta'_{cig} = \mathcal{S}(\text{Logit}(\beta_c) + e_{gi}) \tag{21}$$

$$e_{gi} \sim \mathcal{N}(0, \varphi) \tag{22}$$

The second model B was fit to data simulated from these models for several arbitrarily chosen parameters φ, and the fits correctly displayed no evidence for the presence of variability in isoform preference (Appendix Figure S5b-d).

1.5 BATBayes2: Clustering of single cells based on polyadenylation site usage

To cluster cells based on polyadenylation site usage, we assume that a global correlation structure exists on the matrix p_{cg}. We initially fitted the second model B ("BATBayes") to the data pooled from all 107 cells, and performed a principal component analysis on the posterior means of p. While this approach allowed us to retrieve a rough population structure (Figure 7b), the ESC-FCS and NSC population were difficult to separate using the first two principal components. A possible reason for this relatively weak separation may be that the BATBayes model assumes that no non-random

correlation on p exists between different genes. We therefore extended the second model to explicitly include such a correlation structure on p (see also Figure EV4a), and fit the extended model to the data using the MCMC methodology described above.

In analogy to independent component analysis, we define a score δ_c for each cell and a loading ε_g for each gene. We then allow the mean percentage of major isoform produced (equation 6) to be different for different cells and calculate the expected isoform usage as

$$\rho_{cg} = \mathcal{S}(\rho'_g + \delta_c \cdot \varepsilon_g) \tag{23}$$

We replace the prior in equation 8 by

$$\rho'_g \sim \text{Unif}(-10, 10) \tag{24}$$

The magnitude of the correlation on p is unknown, and determined by the product $\delta \cdot \varepsilon$. Priors on δ and ε therefore cannot be uniquely identified. We fix the prior on δ:

$$\delta_c = \mathcal{N}(0, 1) \tag{25}$$

And estimate the prior on ε_g empirically

$$\varepsilon_g = \mathcal{N}(0, \sqrt{1/\theta}) \tag{26}$$

with hyperprior

$$\theta \sim \exp(0.1) \tag{27}$$

To make sure that this approach does indeed cluster only based on 3' UTR usage and does not require differences in gene expression levels to work, we simulated a mixed population, where the parameters governing mRNA levels (μ and q) were taken from the fit of BATBayes to the NSC population for half of the cells and from the fit to the ESC-2i population for the other half. The parameter governing isoform ratios (ρ) was taken from the fit to the ESC-2i population for all cells. Evidently, cells clustered apart using hierarchical clustering on downsampled gene expression level [2], but not using BATBayes2 (Figure EV4b). We then simulated a population taking μ and q from the fit to the ESC-2i population for all cells, whereas ρ was taken from the ESC-2i population for half of the cells and from the NSC population for the other half. Cells could not be distinguished based on gene expression levels, but isoform-based clustering separated populations very well (Figure EV4b).

The BATBayes2 source code is given in Code EV 2.

References

[1] S. P. B. Brooks and A. G. Gelman. General Methods for Monitoring Convergence of Iterative Simulations. *Journal of Computational and Graphical Statistics*, 7(4):434–455, 1998.

[2] D. Jaitin, E. Kenigsberg, H. Keren-Shaul, N. Elefant, F. Paul, I. Zaretsky, A. Mildner, N. Cohen, S. Jung, A. Tanay, and I. Amit. Massively parallel single-cell RNA-seq for marker-free decomposition of tissues into cell types. *Science*, 343:776–779, 2014.

[3] M. Plummer. JAGS: A Program for Analysis of Bayesian Graphical Models Using Gibbs Sampling. In *Proc 3rd Intl Workshop on Distributed Statistical Computing*, 2003.

[4] M. Plummer, N. Best, K. Cowles, and K. Vines. CODA: Convergence Diagnosis and Output Analysis for MCMC. *R News*, 6(1):7–11, Dec. 2006.

[5] A. Raj and A. V. Oudenaarden. Nature, Nurture, or Chance: Stochastic Gene Expression and Its Consequences. *Cell*, 135:216–226, 2008.

[6] D. J. Spiegelhalter, N. G. Best, B. P. Carlin, and A. van der Linde. Bayesian measures of model complexity and fit. *Journal of the Royal Statistical Society: Series B (Statistical Methodology)*, 64(4):583–639, Oct. 2002.

2 Appendix Figures S1-7

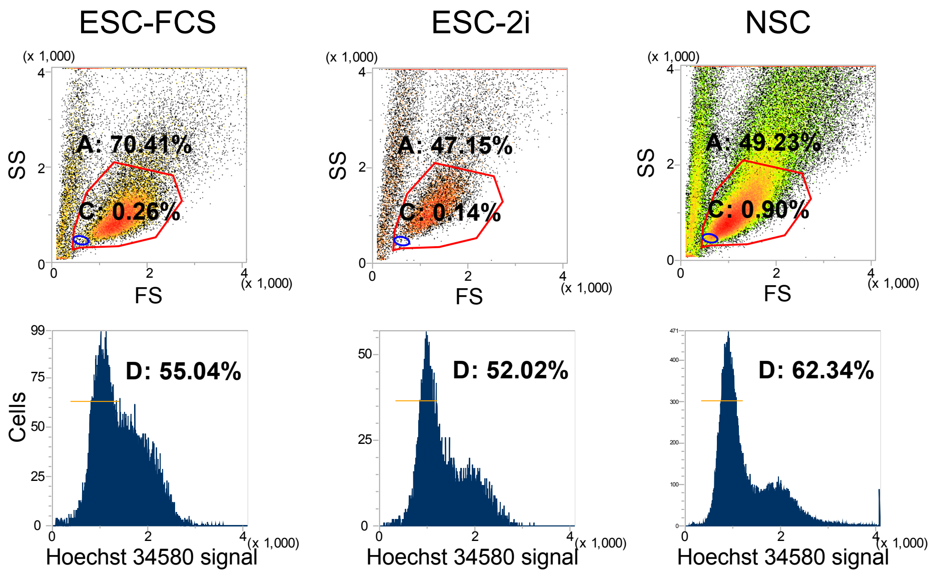

Appendix Figure S 1: Gating strategy during FACS sorting. Cells were stained with Hoechst 34580 and sorted to include only small cells (gate C, top) with 1N chromosome set (gate D, bottom).

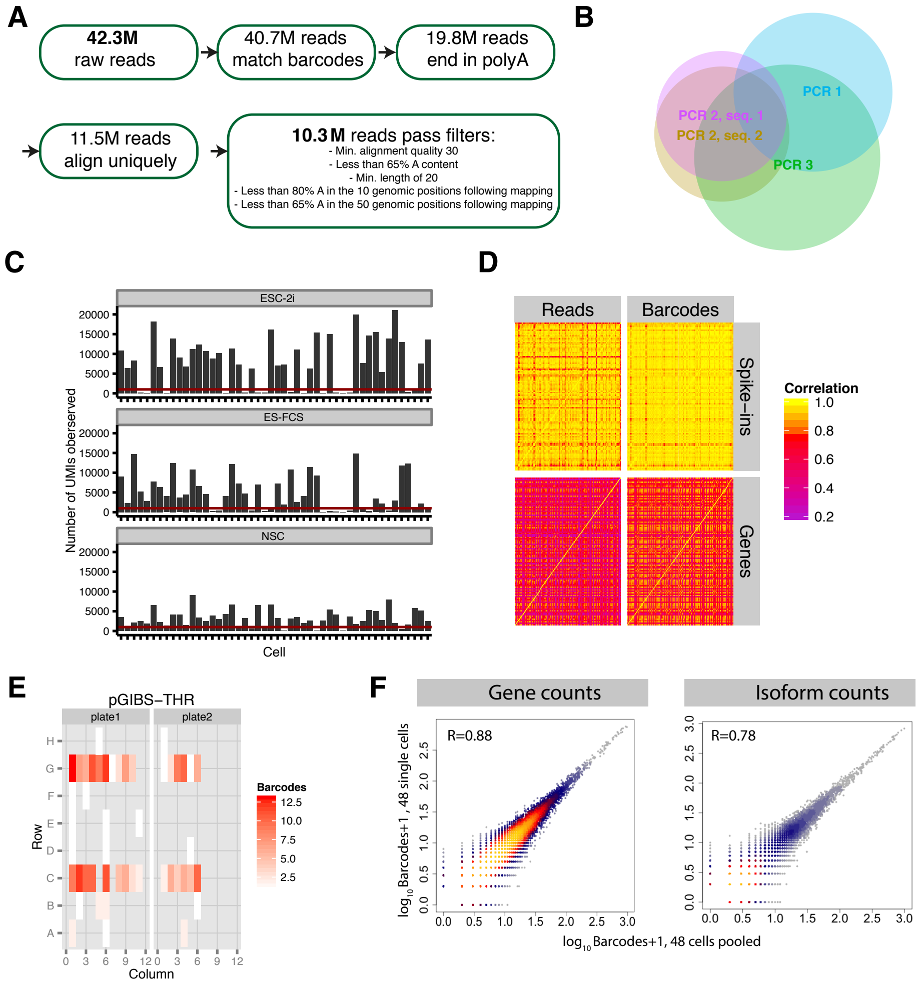

Appendix Figure S 2: Processing of raw reads.

a *Overview of filtering strategy.* In total, 42.3 million reads were obtained, of which 10.3 million reads passed quality control filters.

b *Venn diagram of unique molecules (UMI-cell-gene combinations) identified during four MiSeq runs.* After constructing sequencing libraries on magnetic beads, PCR enrichment was performed prior to sequencing like in standard library preparation protocols. Sequencing deeper into a library did not yield higher coverage (PCR 2, seq. 1 and 2) whereas repeating the final enrichment PCR did increase coverage.

c *Distribution of observed UMIs across cells.* In total, 869.000 unique molecules were observed.

d *Correlations of read and molecule counts.* Each row/column of the heatmaps corresponds to a single cell.

e *Use of a well-specific spike-in reveals that template switching occurs at very low frequencies.* A concern in UMI-based protocols is template switching during PCR, which would result in single original molecules being represented by multiple UMIs in the final sequencing data. In our protocol, such template switches would not only affect the UMI, but also the cell barcode; we therefore included an additional synthetic RNA spike-in (pGIBS-THR) in rows C & G before cell lysis. As multiple rows were pooled prior to PCR, a switch in barcode would result in pGIBS-THR reads in other rows.

f *Alternative strategy of simulating the experiment shown in Figure 2d.* Here, true expression values were estimated by dividing the measured gene expression values from the bulk experiment by the capture efficiency estimate. Obtained correlations are quantitatively identical to the correlations shown in Figure 2d.

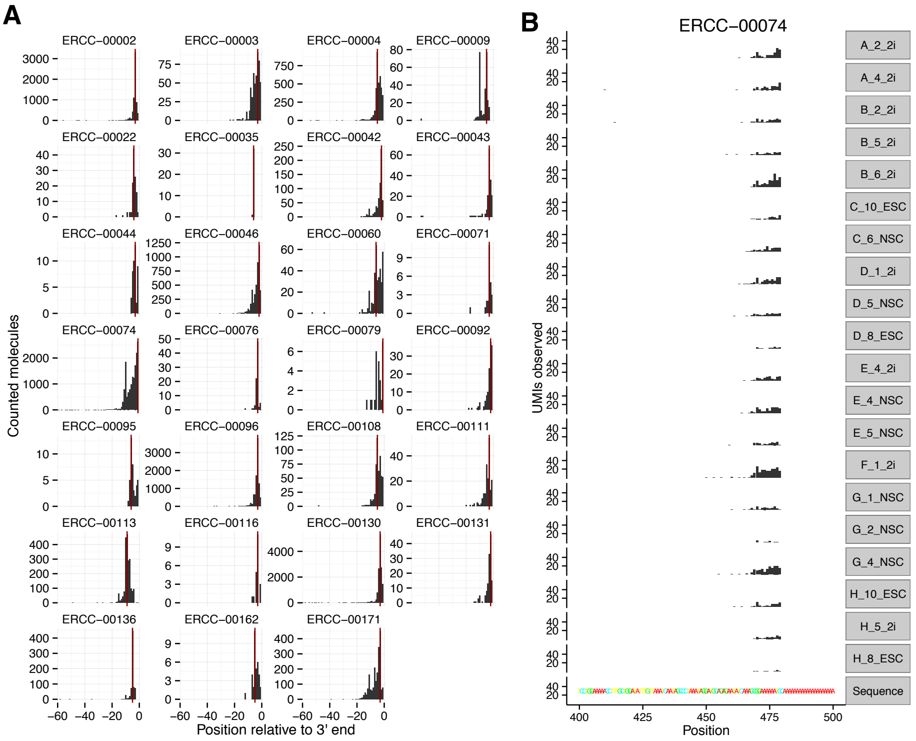

Appendix Figure S 3: Polyadenylation site mapping of control in vitro transcript (IVT) spike ins.

a *Pooled data for all IVTs detected.* Shown are mapped polyadenylation sites of 27 polyadenylated IVT spike-ins with known poly-A site (0). Mappings did not deviate from the true poly-A site by more than 12 bases. Therefore, for all genes, mapping positions within a window of 12 bases were collapsed (red vertical lines).

b *Single-cell data for a sample IVT.* Alignment to a single spike (ERCC-00074) across 20 randomly selected single cells is shown. No obvious biases across single cells were apparent.

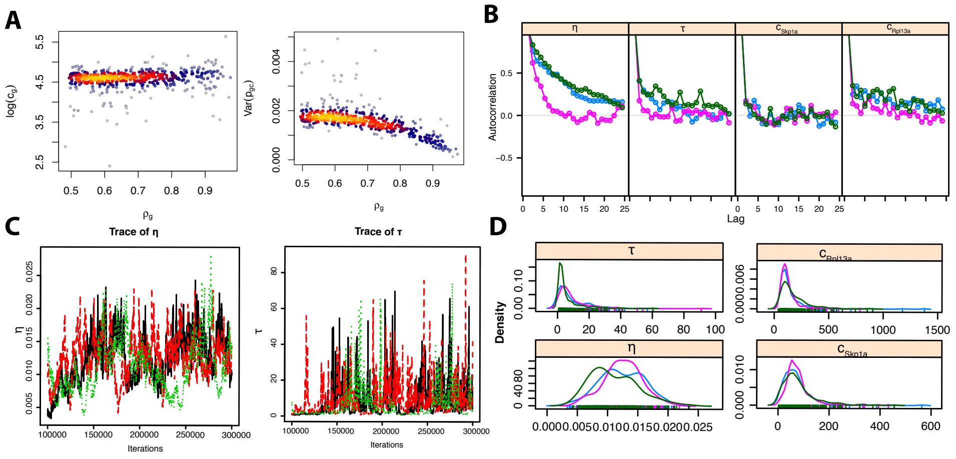

Appendix Figure S 4: Model fitting based on Monte Carlo Markov Chains.

a *Choice of parametrization.* The Beta distribution governing isoform preference was parameterized by a concentration parameter instead of a variance because the concentration parameter is independent of the mean.

b *Convergence diagnostics for a typical Monte Carlo Markov Chain*, the second model fitted to ESC-2i data. Here, three randomly initialized chains were run for 300.000 iterations each, a burn-in of 100.000 iterations was discarded and a thinning interval of 300 was applied. For the hyperparameters η and τ, as well as for randomly selected gene-wise isoform variability parameters c, the plots display autocorrelation *(b)*, trace *(c)* and density *(d)*.

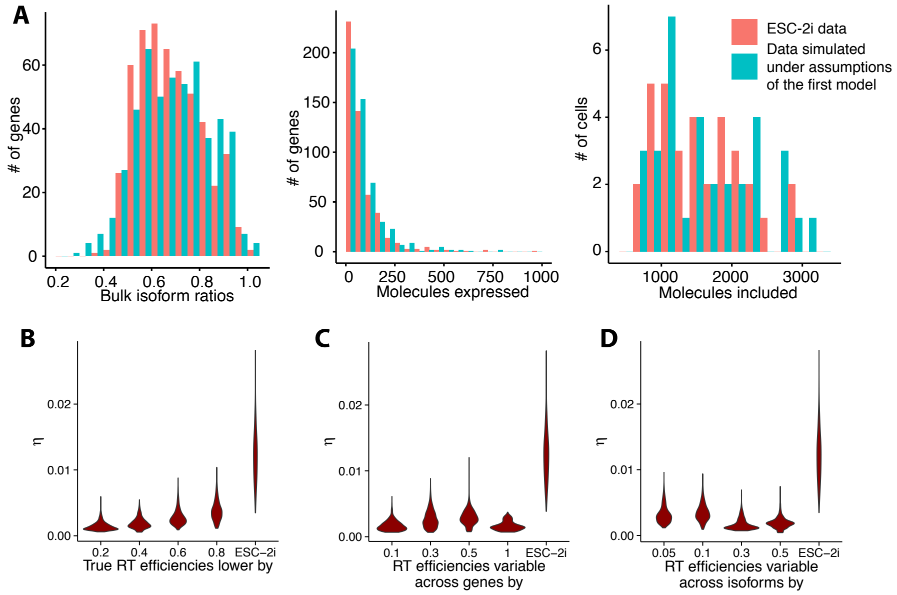

Appendix Figure S 5: Simulations to assess model sensitivity to input parameters.

a *Simulations recapitulate data.* Distributions of bulk isoform ratios, gene expression levels and molecular counts estimated from the ESC-2i data and for a data set that was simulated using the assumption of no variability in isoform preference (First Model).

b *Model output is robust to changes in the estimated capture efficiency.* Posterior densities of variability in isoform preference η for the second model fit to data sets simulated using the assumption that isoform preference is identical in all single cells, and that the BATSeq capture efficiencies were not measured correctly. In *b)* true RT efficiencies were assumed to be lower than the estimated value by a factor between 0.2 and 0.8. In c), capture efficiencies were assumed differ for different genes by a standard deviation between 0.1 and 1. In *d)*, RT capture efficiencies were assumed differ for different genes and isoforms by a standard deviation between 0.05 and 0.5. The posterior density of the fit to the ESC-2i data was included for reference.

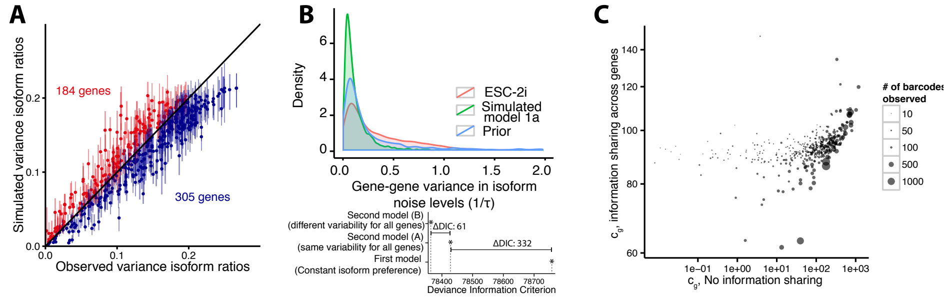

Appendix Figure S 6: Model Comparison.

a *A frequentist approach to single-cell isoform analysis.* For each gene, the observed variance in isoform ratios (x-axis) was plotted against the variance obtained by simulating the first model (isoform preference equal in all cells). Points (y-axis) denote medians, error bars denote the interquartile range. 1000 simulations were run for each gene. For genes below the diagonal (blue), the observed variance exceeds what is expected from simulations, for genes above the diagonal (red) the observed variance is smaller than expected from simulations.

b *Different genes differ slightly in isoform noise level.* Comparison of model A (isoform preference variability is equal in different genes) and model B (isoform preference variability is different in different genes). Upper panel: Posterior densities of the gene-gene variance in isoform noise levels ($1/\tau$) for the ESC-2i data (red) and a dataset that was simulated under the assumptions of model A(green). The prior is also shown (blue). Lower panel: Comparison of model A and model B by the DIC.

c *Information sharing across genes leads to moderated estimates of isoform preference variation in the limit of data.* Concentration parameters c were once estimated for all genes individually (x-axis) and once for all genes in parallel, assuming an empirically estimated log-normal prior. For genes with extremely sparse data, the model frequently returned very low estimates of c when no information sharing was applied.

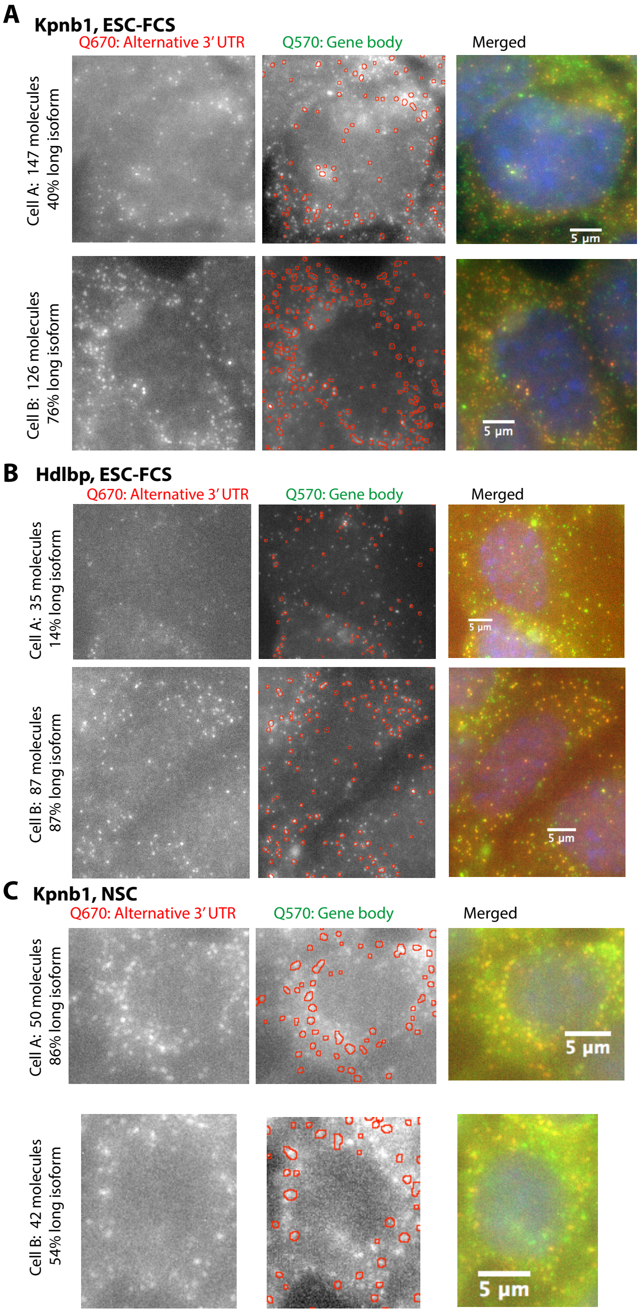

Appendix Figure S 7: Raw smFISH data for additional genes. As in Main Figure 6, dots from the alternative 3' UTR channel are superimposed on the gene body channel to demonstrate colocalization.

3 Appendix Table S1: List of buffers used for BAT-Seq

For primer sequences, see Appendix Table S2. All volumes given in this table are sufficient to process one 96-well plate of cells.

Single-Cell lysis buffer	
10% NP-40	10 μL
RNAsin plus (Promega, $40U/\mu L$)	$5\mu L$
RNAse-free water	$135\mu L$
ERCC Spike-In Mix, 1:1.000.000 diluted (Invitrogen)	50 μL
Priming master mix	
10x PCR Buffer w/ 15mM MgCl (Applied Biosystems)	$165\mu L$
10 mM dNTPs (NEB)	5.5 μL
RNAsin plus (Promega, $40U/\mu L$)	$110\mu L$
RNase-free water	764.5 μL
Priming buffer	
Priming master mix	$9.5\mu L$
$0.8333\mu M$ early multiplexing primer eaMPX	$0.5\mu L$
Barcoding RT buffer	
10x PCR Buffer w/ 15mM $MgCl_2$ (Applied Biosystems)	15 μL
0.1M DTT (Invitrogen)	$18.75\mu L$
RNase free water	97.5 μL
SuperScript III (Invitrogen, 200U/μL)	$18.75\mu L$
ExoI buffer	
NEBuffer 2 (NEB)	$12\mu L$
Exonuclease I (NEB, 20U/μL)	$9\mu L$
RNase free water	$99\mu L$
PolyA tailing buffer	
10x PCR Buffer w/ 15mM $MgCl_2$ (Applied Biosystems)	$25\mu L$
100mM dATP	$7.5\mu L$
RNASeq H (Invitrogen, 2U/μL)	$6\mu L$
TdT Enzyme (Roche, 400U/μL)	$20.4\mu L$
RNase free water	$190\mu L$
PCR mix I	
2x Terra Direct Buffer (Clontech)	$612.5\mu L$
10M Tagging primer	$7\mu L$
Terra DNA polymerase	$50\mu L$
Water	$205.5\mu L$
PCR mix II	
2x Terra Direct Buffer	$616\mu L$
100M PCR Primer	$23.8\mu L$
Water	$606\mu L$
Elution buffer	
10mM Tris-HCl in water, pH 7.5, filtered through $0.22\mu M$ pores	
IVT mix	
10x Transcription Reaction Buffer (Roche)	$39\mu L$
10mM NTP (Invitrogen)	$39\mu L$
T7 RNA Polymerase (Roche, 20U/μL)	$19.5\mu L$

RNase-free water	19.5μL
RNAsin plus (Promega)	13μL
RNA fragmentation buffer	
200mM Tris Acetate, pH 8.1	
500nM KOAc	
150mM MgOAc	
Library RT buffer	
5x First strand buffer (Invitrogen)	20μL
0.1M DTT (Invitrogen)	10μL
Actinomycin D (1.25mg/mL)	1.5μL
RNAsin plus (Promega)	2.5μL
Superscript II (Invitrogen, 200U/μL)	2.5μL
Second Strand buffer	
10x DNA polymerase I buffer (Fermentas)	25μL
10mM dNTP	12.5μL
RNase H (2U/μL, Invitrogen)	2.5μL
DNA polymerase I (Fermentas, 10U/μL)	10μL
2x B&W buffer	
10mM Tris-HCl, pH 7.5	
1mM EDTA	
2M NaCl	

4 Appendix Table S2: List of primers used for BATSeq

eaMPX_A	TATAGAATTCGCGGCCGCTCGCGATCACTGTNNNNNNNNVTTTTTTTTTTTTTTTTTTTTVN
eaMPX_B	TATAGAATTCGCGGCCGCTCGCGATATTCCGNNNNNNNNVTTTTTTTTTTTTTTTTTTTTVN
eaMPX_C	TATAGAATTCGCGGCCGCTCGCGATGCTACCNNNNNNNNVTTTTTTTTTTTTTTTTTTTTVN
eaMPX_D	TATAGAATTCGCGGCCGCTCGCGATCGAAACNNNNNNNNVTTTTTTTTTTTTTTTTTTTTVN
eaMPX_E	TATAGAATTCGCGGCCGCTCGCGATAGCGCTNNNNNNNNVTTTTTTTTTTTTTTTTTTTTVN
eaMPX_F	TATAGAATTCGCGGCCGCTCGCGATGTATAGNNNNNNNNVTTTTTTTTTTTTTTTTTTTTVN
eaMPX_G	TATAGAATTCGCGGCCGCTCGCGATTCCGTCNNNNNNNNVTTTTTTTTTTTTTTTTTTTTVN
eaMPX_H	TATAGAATTCGCGGCCGCTCGCGATGAATGANNNNNNNNVTTTTTTTTTTTTTTTTTTTTVN
eaMPX_I	TATAGAATTCGCGGCCGCTCGCGATATAGATNNNNNNNNVTTTTTTTTTTTTTTTTTTTTVN
eaMPX_J	TATAGAATTCGCGGCCGCTCGCGATATCGTGNNNNNNNNVTTTTTTTTTTTTTTTTTTTTVN
eaMPX_K	TATAGAATTCGCGGCCGCTCGCGATCTGATCNNNNNNNNVTTTTTTTTTTTTTTTTTTTTVN
eaMPX_L	TATAGAATTCGCGGCCGCTCGCGATCATTCANNNNNNNNVTTTTTTTTTTTTTTTTTTTTVN
eaMPX_M	TATAGAATTCGCGGCCGCTCGCGATAGTCTTNNNNNNNNVTTTTTTTTTTTTTTTTTTTTVN
eaMPX_N	TATAGAATTCGCGGCCGCTCGCGATTTGCGGNNNNNNNNVTTTTTTTTTTTTTTTTTTTTVN
eaMPX_O	TATAGAATTCGCGGCCGCTCGCGATTGTGCCNNNNNNNNVTTTTTTTTTTTTTTTTTTTTVN
eaMPX_P	TATAGAATTCGCGGCCGCTCGCGATTTCGAANNNNNNNNVTTTTTTTTTTTTTTTTTTTTVN
eaMPX_Q	TATAGAATTCGCGGCCGCTCGCGATGAGAGTNNNNNNNNVTTTTTTTTTTTTTTTTTTTTVN
eaMPX_R	TATAGAATTCGCGGCCGCTCGCGATCGTACGNNNNNNNNVTTTTTTTTTTTTTTTTTTTTVN
eaMPX_S	TATAGAATTCGCGGCCGCTCGCGATCTCTACNNNNNNNNVTTTTTTTTTTTTTTTTTTTTVN
eaMPX_T	TATAGAATTCGCGGCCGCTCGCGATGCTGTANNNNNNNNVTTTTTTTTTTTTTTTTTTTTVN
eaMPX_U	TATAGAATTCGCGGCCGCTCGCGATGGAACTNNNNNNNNVTTTTTTTTTTTTTTTTTTTTVN
eaMPX_V	TATAGAATTCGCGGCCGCTCGCGATTCTGAGNNNNNNNNVTTTTTTTTTTTTTTTTTTTTVN
eaMPX_W	TATAGAATTCGCGGCCGCTCGCGATCCACTCNNNNNNNNVTTTTTTTTTTTTTTTTTTTTVN
eaMPX_X	TATAGAATTCGCGGCCGCTCGCGATTCGGGANNNNNNNNVTTTTTTTTTTTTTTTTTTTTVN
Tagging primer	TATAGAATTCGCGGCCGCTCGCGATAATACGACTCACTATAGGGCGTTTTTTTTTTTTTTTTTTTTTT
PCR primer	GTATAGAATTCGCGGCCGCTCGCGAT
BATSeq capture primer	[Btn]AATGATACGGCGACCACCGAGATCTACACTATAGAATTCGCGGCCGCTCGCGAT

P7_T1_Mpx1§	Fwd: CAAGCAGAAGACGGCATACGAGATCGGTCTCGGCATTCCTGCTGAACCGCTCTTCCGATCTCGTGAT*T<br>Rev: [Phos]ATCACGAGATCGGAAGAGCGGTTCAGCAGGAATGCCGAGACCGATCTCGTATGCCGTCTTCTGCTTG
P7_T1_Mpx2§	Fwd: CAAGCAGAAGACGGCATACGAGATCGGTCTCGGCATTCCTGCTGAACCGCTCTTCCGATCTAAGCTA*T<br>Rev: [Phos]TAGCTTAGATCGGAAGAGCGGTTCAGCAGGAATGCCGAGACCGATCTCGTATGCCGTCTTCTGCTTG
P7_T1_Mpx3§	Fwd: CAAGCAGAAGACGGCATACGAGATCGGTCTCGGCATTCCTGCTGAACCGCTCTTCCGATCTGTAGCC*T<br>Rev: [Phos]GGCTACAGATCGGAAGAGCGGTTCAGCAGGAATGCCGAGACCGATCTCGTATGCCGTCTTCTGCTTG
P7_T1_Mpx4§	Fwd: CAAGCAGAAGACGGCATACGAGATCGGTCTCGGCATTCCTGCTGAACCGCTCTTCCGATCTTACAAG*T<br>Rev: [Phos]CTTGTAAGATCGGAAGAGCGGTTCAGCAGGAATGCCGAGACCGATCTCGTATGCCGTCTTCTGCTTG
P7_T1_Mpx5§	Fwd: CAAGCAGAAGACGGCATACGAGATCGGTCTCGGCATTCCTGCTGAACCGCTCTTCCGATCTACATCG*T<br>Rev: [Phos]CGATGTAGATCGGAAGAGCGGTTCAGCAGGAATGCCGAGACCGATCTCGTATGCCGTCTTCTGCTTG
P7_T1_Mpx6§	Fwd: CAAGCAGAAGACGGCATACGAGATCGGTCTCGGCATTCCTGCTGAACCGCTCTTCCGATCTGCCTAA*T<br>Rev: [Phos]TTAGGCAGATCGGAAGAGCGGTTCAGCAGGAATGCCGAGACCGATCTCGTATGCCGTCTTCTGCTTG
P7_T1_Mpx7§	Fwd: CAAGCAGAAGACGGCATACGAGATCGGTCTCGGCATTCCTGCTGAACCGCTCTTCCGATCTTGGTCA*T<br>Rev: [Phos]TGACCAAGATCGGAAGAGCGGTTCAGCAGGAATGCCGAGACCGATCTCGTATGCCGTCTTCTGCTTG
PE2.0	CAAGCAGAAGACGGCATACGAGATCGGTCTCGGCATTCCTGCTGAACCGCTCTTCCGATCT
PE1.BATSeq	AATGATACGGCGACCACCGAGATCTACACTATAGAATTCGCGGCCGCTCGCGAT
BATSeq first read sequencing primer	TATAGAATTCGCGGCCGCTCGCGAT

[Btn]: 5' Biotinylation
[Phos]: 5' Phosphorylation
*: S-linkage between the two bases
§: To create double-stranded P7_T1_Mpx linkers from forward and reverse oligos, mix oligos at $2.5\mu M$ in the presence of 40mM Tris-HCl pH 7.5 and 50mM NaCl; Incubate sample for 5min at 95°C and let it cool to 65°C at a cooling rate of 0.1°C/s; incubate for 5min at 65C and let cool to 4C at a rate of 0.1 °C. Linkers are stored at -20 °C and thawed on ice to prevent denaturation.
