# Supplementary material for: Single-cell polyadenylation site mapping reveals 3′ isoform choice variability
Source: Mol Syst Biol. 2015 Jun 3;11(6):812. doi: 10.15252/msb.20156198 (PMC4501847; doi:10.15252/msb.20156198)
Supplement: Supplementary file 2 [file msb0011-0812-sd2.pdf]

Expanded View Figures

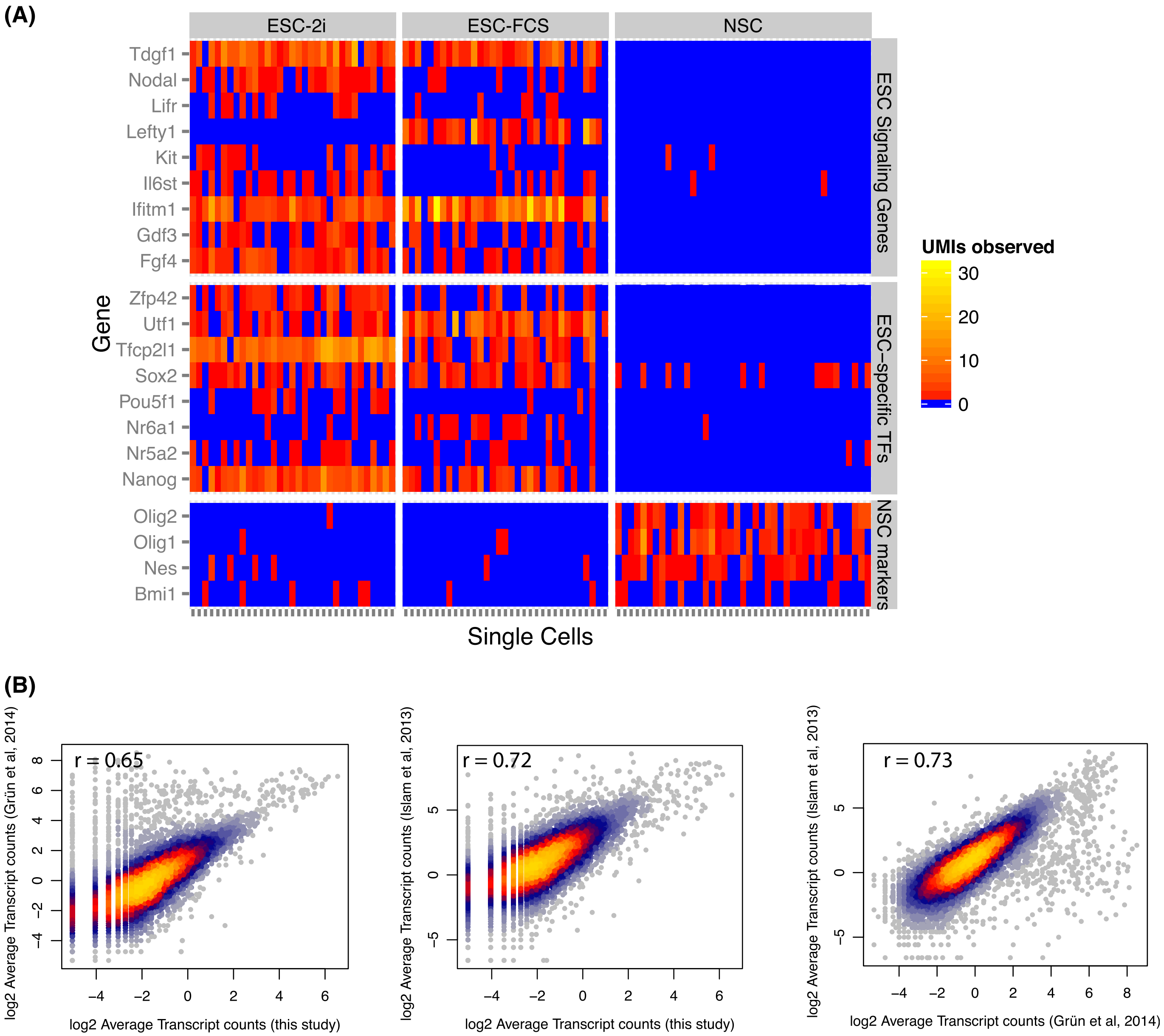

Figure EV1. Gene expression in single ESC-2i, ESC-FCS, and NSC cell populations follows expected patterns.

A Expression patterns of ES- and NS-marker genes in single cells in the three populations

B Correlation of average transcript counts with previously published studies. Per-cell observed average transcript counts measured in ESC-FCS in this study were plotted against per-cell observed average transcript counts measured in two recent studies that also performed single-cell transcriptomics of embryonic stem cells (Islam *et al*, 2013; Grün *et al*, 2014). In the rightmost panel, data from these two studies were compared.

© 2015 The Authors

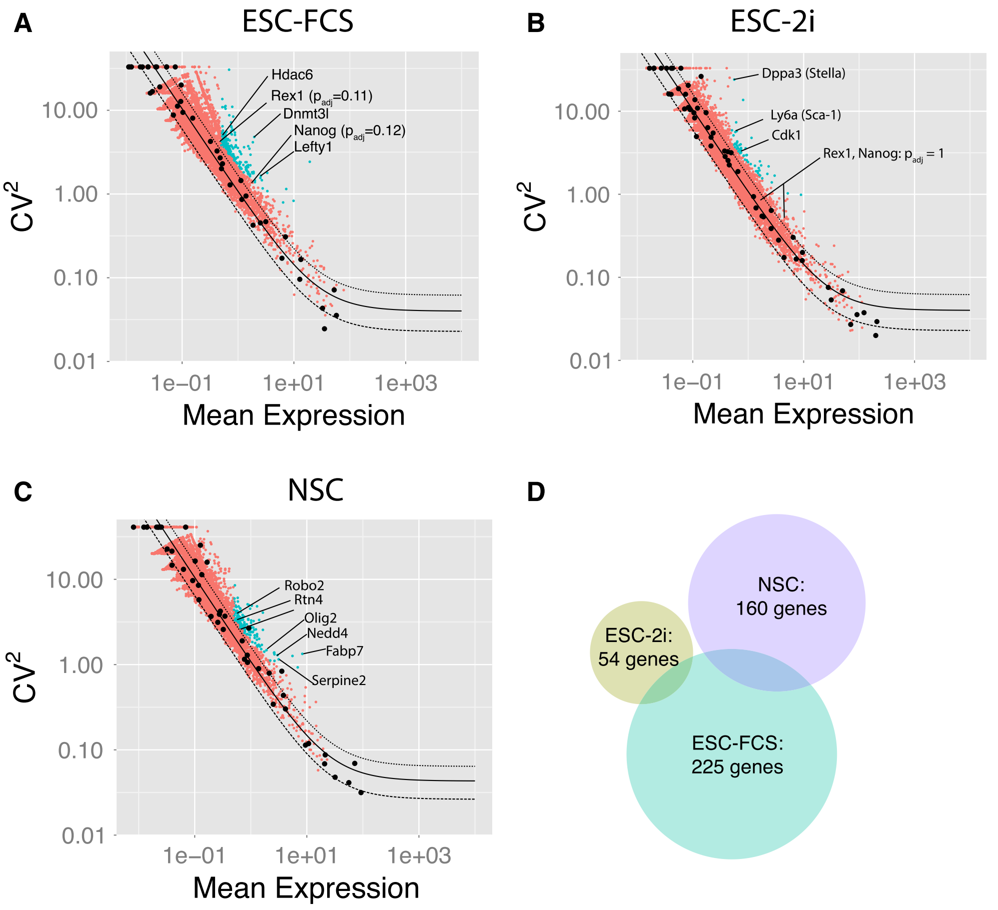

Figure EV2. Genes of variable expression in homogeneous stem cell populations.

A–C Identification of variably expressed genes. Variance of observed spike-in counts (black dots) was used to estimate expected technical noise (Brennecke *et al*, 2013) within the ESC-FCS (A), ESC-2i (B), and NSC (C) populations. Blue dots represent genes for which the observed variance exceeds what is expected from technical noise with a FDR-adjusted *P*-value of < 0.1. For a table of all genes and their *P*-values for heterogeneous expression, see Table EV1.

D Venn diagram of the number of genes identified.

 © 2015 The Authors

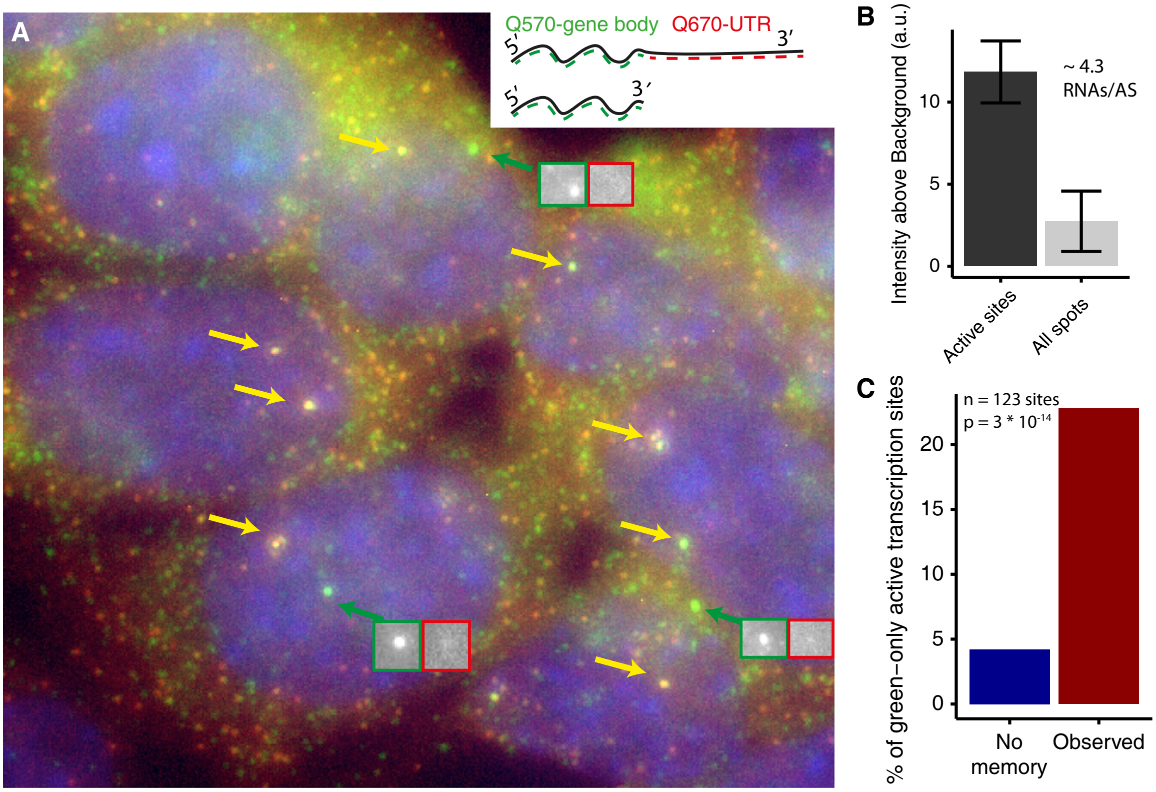

Figure EV3. Single isoforms dominate active sites of transcription.

A While most active sites of transcription of the *Kpnb1* gene contain both red (alternative UTR) and green (common sequence) signal, some are green only (red and green channel are shown in insets). Shown is a field of view of ESC-FCS cells.

B Quantification of spot brightness to estimate the number of mRNA molecules per active site of transcription. Error bars denote standard deviation.

C The observed prevalence of green-only active sites is contrasted to the prevalence obtained when assuming random isoform choice during each cycle of transcription. *P*-value is from a binomial test.

© 2015 The Authors

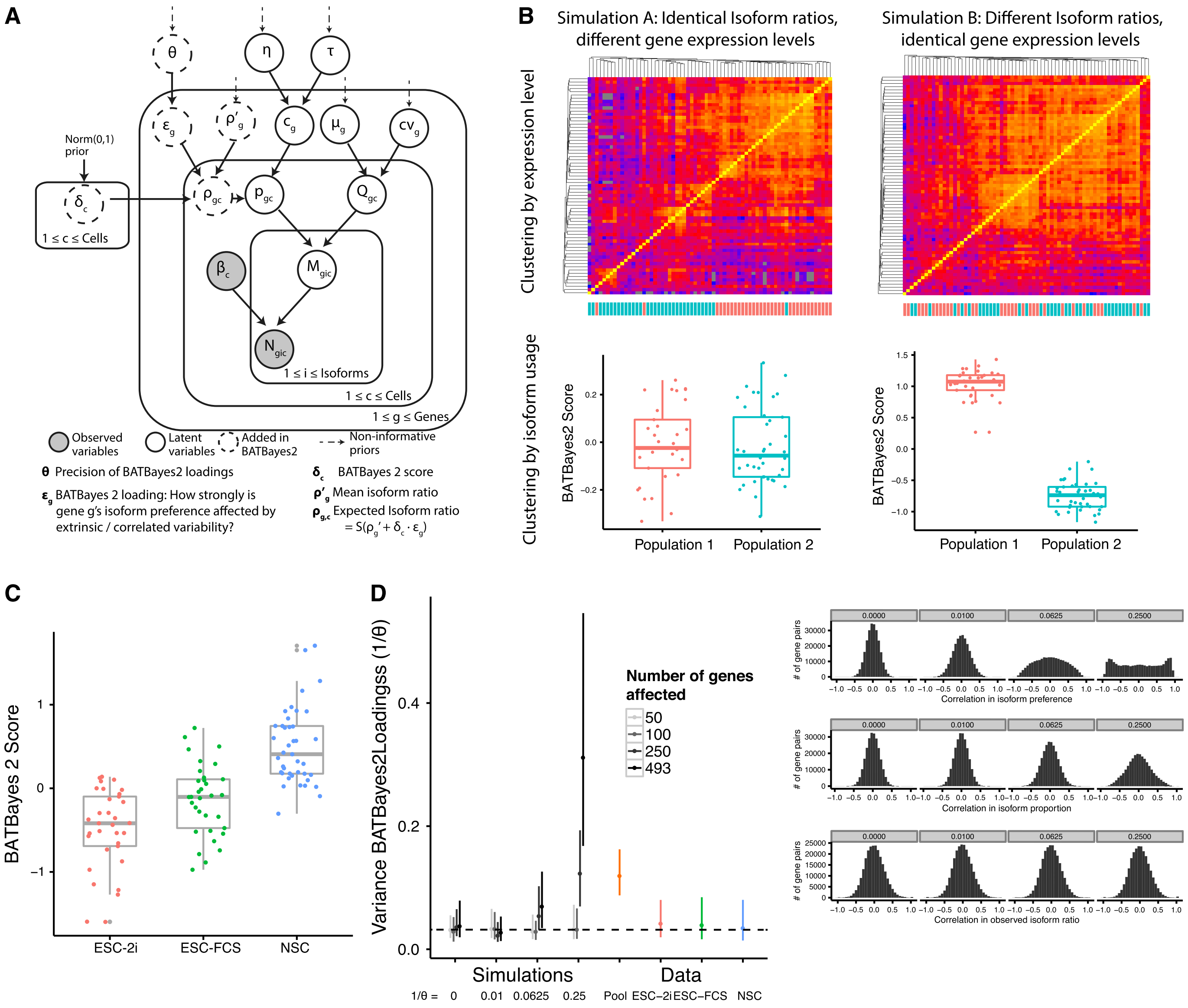

Figure EV4. BATBayes2, a model for correlated variability in 3′ isoform usage.

A Directed acyclical graph of the BATBayes2 model. To model global, correlated changes in 3′ isoform usage across single cells, each cell was assigned a score and each gene was assigned a loading. The effect of correlated variability on a given gene in a given cell was obtained by multiplying score with loading.

B Simulations. To show that BATBayes2 is affected by isoform ratios, but not by gene expression levels, populations were simulated where expression levels were equal in the two populations but isoform usage differed (scenario A) or vice versa (scenario B). Cells were clustered using hierarchical clustering of gene expression levels (upper panels) or BATBayes2 (lower panels).

C Clustering based on isoform preference using only genes with < 2-fold difference in expression level between any two developmental stages. Of the 493 genes used originally, only 88 were used in creating this figure.

D No evidence for correlated isoform choice variability within homogeneous populations. The BATBayes2 model was fit to data from the individual cell populations separately (red, green and blue dots), as well as to data from all cells pooled. While the variance of BATBayes loadings (*y*-axis, conceptually similar to the variance explained by the first principle component, see also Appendix Supplementary Text) was clearly above background for the pooled cell population (orange), no evidence for coordinated variability was found for the individual cell populations (red, green and blue). We then used simulations for populations in which different numbers of genes (different shades of gray) were affected by correlated variability with set variance. We found that the model is only able to detect relatively strong correlations that affect at least half of the genes under study. To explain why, we plotted distributions of gene-pair correlations for different strength of "extrinsic" variability (right panel, left to right). While correlations at the level of the non-observed variable isoform preference are clearly visible also at weaker extrinsic noise strength (top panel), following random partitioning (middle panel) and technical noise (bottom panel), correlations in the raw data are barely apparent even at very strong extrinsic variability.

 © 2015 The Authors
