# Supplementary material for: Single-cell polyadenylation site mapping reveals 3′ isoform choice variability
Source: Mol Syst Biol. 2015 Jun 3;11(6):812. doi: 10.15252/msb.20156198 (PMC4501847; doi:10.15252/msb.20156198)
Supplement: Supplementary file 7 [file msb0011-0812-sd7.pdf]

Single cell polyadenylation site mapping reveals 3' isoform choice variability

Lars Velten, Simon Anders, Aleksandra Pekowska, Aino I Järvelin, Wolfgang Huber, Vicent Pelechano and Lars M Steinmetz

Corresponding author: Lars M Steinmetz EMBL, Genome Biology Unit

Review timeline:

Received:	9 September 2014
Editorial Decision:	19 October 2014
Re-submission:	11 December 2014
Editorial Decision:	15 February 2015
Re-submission:	26 March 2015
Editorial Decision:	22 April 2015
Revision received:	24 April 2015
Accepted:	03 May 2015

Editor: Thomas Lemberger

Transaction Report:

(Note: With the exception of the correction of typographical or spelling errors that could be a source of ambiguity, letters and reports are not edited. The original formatting of letters and referee reports may not be reflected in this compilation.)

1st Editorial Decision 19 October 2014

Thank you again for submitting your work to Molecular Systems Biology. We have now heard back from the referees whom we asked to evaluate your manuscript. As you will see from the reports below, the referees raise substantial concerns on your work, which, I am afraid to say, preclude its publication.

The reviewers acknowledge the value of your approach and of the presented methodology. However, beside several technical issues, both reviewers note that the lack of follow up represents a clear limitation of the study. Thus, the main findings are not replicated with an orthogonal assay and the biological insights provide by the analysis remain limited. We have also briefly consulted with a member of our advisory board who also acknowledged the value of the BATSeq and BATBayes methods but noted that orthogonal validation and follow up were lacking.

As such, I am afraid we are not convinced that the study would provide the kind of insights or the level of conclusiveness our audience would expect in Molecular Systems Biology

Nevertheless, the editor and the reviewers expressed interest in the subject matter and your approach, and we would not be opposed to consider a new submission that extends this work, provided convincing orthogonal validation can be provided and deeper biological insights would result from the analysis.
This new submission would have a new number and receipt date. We recognise that this may involve further experimentation and analysis, and we can give no guarantee about its eventual acceptability. However, if you do decide to follow this course then it would be helpful to enclose

© European Molecular Biology Organization

with your re-submission an account of how the work has been altered in response to the points raised in the present review.

I am sorry that the review of your work did not result in a more favourable outcome on this occasion, but I hope that you will find the reviews below useful.

Reviewer #1:

The authors modify existing single cell RNA-seq protocols in order to identify polyadenylation sites at single molecule resolution. They also introduce a novel statistical framework to interpret this type of data that explicitly models two sources of non-regulated noise, and apply these techniques to mESC in two different culture conditions, as well as a set of NPCs. They find that developmental progression is positively correlated with 3' UTR length, intriguingly allowing for cells to be placed into their developmental state exclusively based on polyA site usage. Moreover, they find that even within a particular stage, there is a greater variability in polyadenylation across single cells than can be explained by technical noise or chance alone.

The work presented here is creative and valuable to the field. While there has been extensive discussion and methodological development surrounding heterogeneity in gene expression, the strength of the manuscript is the robust statistical framework described for studying post-transcriptional regulation of RNA. The method clearly describes two source of 'non-regulated' variability (technical and random partitioning), and accurately models these to identify genes exhibiting biological variation in polyadenylation site selection. The statistical modeling is elegant, and the simulations and data analysis are well-performed and convincing.

There are limitations to the study as well. Alternative experimental validation (i.e. RNA FISH) would have been highly valuable, particularly for genes where the authors' observed particularly high levels of single cell heterogeneity for 3' UTR usage. Moreover, the characterization of cellular heterogeneity in 3' UTR usage, while novel, is somewhat underdeveloped. It is unclear if this heterogeneity represents stochastic differences, or if there is structure in this heterogeneity (i.e. coherent modules of genes that show longer/shorter UTR usage across subpopulations of mESC). While identifying such modules would be very exciting, it may be beyond the scope of this study.

Overall I believe that the ideas and analytical tools presented in this work represent an important contribution. However, there are a few major issues below that I believe the authors need to (and should) be able to address.

1. BATSeq method for mapping/quantification of polyA sites

- While UMIs are becoming commonplace for single cell methods, one significant challenge with UMI-based protocols is that excess RT primer which remains after reverse transcription can also serve as a primer during PCR (see Islam et al 2013, Soumillion et al 2014 Biorxiv). When this happens, there is a 'barcode switch' - i.e. a new UMI is introduced during PCR, which means that a single original molecule can be represented by multiple UMIs in the final sequencing data. This is not an issue for non-PCR based amplification methods (Grun et al 2014, Jaitlin et al 2013), but the authors here do PCR cycles prior to T7 amplification, so this is a significant concern.

The published literature to date suggests two ways around this problem. The first is to do an exonuclease digestion after RT to remove excess RT primer (Soumillion et al 2014), or to place the UMI on the 5' end using an RNA Template Switch Oligo, which hydrolyzes during PCR annealing (Islam et al 2013). However, absent these or similar steps, the UMI tags from BatSeq do not truly represent unique molecules.

To ensure that the biological conclusions are accurate, the authors should generate additional data with an exonuclease cleanup step (or similar) to address this concern. Even a small number of cells (~8-10) would be sufficient to show that heterogeneity in UTR usage still remains after this experimental correction. In the absence of this, I am concerned that PCR noise remains a potential source of noise in the authors' data - which could potentially invalidate some of their biological findings (as PCR noise cannot be controlled for in their statistical model).

- A now standard metric for single cell techniques is to correlate the 'in silico' average of single cells with a population experiment. Did the authors generate population data (i.e. from 48, or even 10,000 pooled mESC). It would be valuable to see the correlation of averaged single cell and population measurements, both for gene expression, and for 3' UTR usage.

2. Cell identity can be inferred on based on 3' UTR usage alone.

This is an interesting result, especially as this inference is performed only based on fractional UTR usage, and does not include gene expression. As an important control, the authors should show that this result holds even when only considering genes that are expressed at similar levels (i.e. average expression within 2 fold) between mESC and NSC. This is to control for the inherent fact (as the authors extensively describe) that noise in UTR estimation (both biological and technical) is highly dependent on expression abundance.

3. Variability in isoform preference across 'homogeneous' cells.

- My understanding is that the BatBayes model only infers a global parameter for heterogeneity in 3' UTR usage, rather than inferring a gene by gene estimate of single cell variation. If this is correct, the authors should state this more clearly in the text.

- It seems possible that a cell may choose to express long isoforms for some genes, and shorter versions of others. Another cell may make completely opposite isoform choices, but both cells would have the same average 3' UTR length. Would BatBayes accurate capture the heterogeneity in this scenario?

- I see that the authors do implement a frequentist control where heterogeneity/significance is calculated on the gene level. However, while there is a significant p-value, the results are not terribly convincing (i.e. not an obvious visual skew below the line in Fig. E7e). Do the authors' observe any functional enrichments for genes that are 'significantly heterogeneous' by this metric, or any correlations in the isoform choice for these genes across single cells?

Reviewer #2:

The manuscript "Single cell polyadenylation site mapping reveals 3' isoform choice variability" by Velten et al., where an approach termed BATseq is employed to perform quantitative polyadenylation analysis in single mouse embryonic and neural stem cells.

Given the effort required to achieve BATseq libraries, I was surprised that the authors only sequenced to a depth of ~40 million reads (of which ~10% were useful). Sequencing the samples to higher depth would likely have revealed considerably more poly-adenylation examples and complexity, and this additional coverage would surely have improved data quality, and the resulting analysis being more robust and increase the many different aspects of the manuscript substantially.

A second broad recommendation would be that the authors, having established a technique and statistical framework for analyzing single-cell poly-adenylation events, should provide further evidence on how this approach provide novel biological insight. For example, what is the variation in the choice of isoforms between multiple cells of a single type, relative to what is the variation between multiple cells of different cell types? Is it possible to identify enriched sequence motifs associated with specific poly-adenylation trends within cell lines etc. ? Is there any correlation between the variation in 3UTR choice and variation in alternative splicing within a gene? Do specific classes of genes or developmental stages show greater variation in polyadenylation? These are some examples of avenues that could be further investigated to provide insight to the mechanisms of polyadenylation.

Finally, I would like to commend the authors on a well-executed study. The manuscripts figures, text and presentation are all clear, well-written and accessible.

Minor Points:
1. Gene names are not italicized on several pages (eg. Page 6 Nanog, Dnmt3l. Lefty1, Stella, and

Sca-1)
2. I found interpreting the paragraph at top of page 7 a little bit confusing. My understanding is that the ESC-2i population display several genes with highly variable polyadenylation. However, the number of identified variable genes were smaller in ESC-2i than in ESC-FCS. Yet the authors confirm that ES maintained in 2i medium do not constitute a completely homogenous population. However, this seems a contradictory, with ESC-2i being more homogenous than ESC-FCS. Could this please be clarified?

Board Advice:

I think the BATSeq and BATBayes are methods they try to establish,which is OK, but there is not enough validation. I find what comes after these methods strangely disconnected, not really followed up, and a bit hanging in the air- don't know how these results can be validated or what kind of prediction could be followed up.

Re-submission 11 December 2014

(see next page)

Reviewer #1:
The authors modify existing single cell RNA-seq protocols in order to identify polyadenylation sites at single molecule resolution. They also introduce a novel statistical framework to interpret this type of data that explicitly models two sources of non-regulated noise, and apply these techniques to mESC in two different culture conditions, as well as a set of NPCs. They find that developmental progression is positively correlated with 3' UTR length, intriguingly allowing for cells to be placed into their developmental state exclusively based on polyA site usage. Moreover, they find that even within a particular stage, there is a greater variability in polyadenylation across single cells than can be explained by technical noise or chance alone.

*The work presented here is **creative** and **valuable** to the field. While there has been extensive discussion and methodological development surrounding heterogeneity in gene expression, the strength of the manuscript is the **robust statistical framework** described for studying post-transcriptional regulation of RNA. The method clearly describes two source of 'non-regulated' variability (technical and random partitioning), and accurately models these to identify genes exhibiting biological variation in polyadenylation site selection. The **statistical modeling is elegant**, and the simulations and data analysis are **well-performed** and **convincing**.*

We thank the reviewer for his encouraging comments and for his insightful, in-depth evaluation of our work.

*There are limitations to the study as well. **Alternative experimental validation** (i.e. **RNA FISH**) would have been highly valuable, particularly for genes where the authors' observed particularly high levels of single cell heterogeneity for 3' UTR usage. Moreover, the characterization of cellular heterogeneity in 3' UTR usage, while novel, is somewhat underdeveloped. It is unclear if this heterogeneity represents stochastic differences, **or if there is structure in this heterogeneity** (i.e. coherent modules of genes that show longer/shorter UTR usage across subpopulations of mESC). While identifying such modules would be very exciting, it may be beyond the scope of this study.*

We added confirmatory RNA FISH data for two genes in two developmental states each to our work (new Figure 5, Figure E8). We further investigated whether modules of genes co-vary within homogeneous cell populations. In detail, we applied the BATBayes2 model, which was originally developed to separate cells from distinct populations based on isoform use, to the data from the individual cell populations. While we cannot exclude that smaller numbers of genes modestly vary in a correlated manner (e.g. due to shared miRNA binding sites), we find no correlated variability of a larger number of genes (new Figure E9d). We therefore conclude that isoform choice variability is likely to be governed by cis-acting factors. In line with that, our new data supports that active sites of transcription are dominated by single mRNA isoforms (new Figure 7), suggesting that polyadenylation sites remain active for several rounds of transcription.

*Overall I believe that the ideas and analytical tools presented in this work represent an **important contribution. However, there are a few major issues below that I believe the authors need to (and should) be able to address**.*

1. BATSeq method for mapping/quantification of polyA sites

*- While UMIs are becoming commonplace for single cell methods, one significant challenge with UMI-based protocols is that excess RT primer which remains after reverse transcription can also serve as a primer during PCR (see Islam et al 2013, Soumillion et al 2014 Biorxiv). When this happens, there is a **'barcode switch'** - i.e. a new UMI is introduced during PCR, which means that a single original molecule can be represented by multiple UMIs in the final sequencing data. This is not an issue for non-PCR based amplification methods (Grun et al 2014, Jaitlin et al 2013), but the authors here do PCR cycles prior to T7 amplification, so this is a significant concern.*

*The published literature to date suggests two ways around this problem. The first is to do an **exonuclease digestion** after RT to remove excess RT primer (Soumillion et al 2014), or to place the UMI on the 5' end using an RNA Template Switch Oligo, which hydrolyzes during PCR annealing (Islam et al 2013). However, absent these or similar steps, the UMI tags from BatSeq do not truly represent unique molecules.*

*To ensure that the biological conclusions are accurate, the authors **should generate additional data with an exonuclease cleanup step** (or similar) to address this concern. Even a small number of cells (~8-10) would be sufficient to show that heterogeneity in UTR usage still remains after this experimental correction. In the absence of this, I am concerned that PCR noise remains a potential source of noise in the authors' data - which could potentially invalidate some of their biological findings (as PCR noise cannot be controlled for in their statistical model).*

We did already use an exonuclease cleanup step in the original BATSeq protocol (Methods, section “cDNA synthesis and amplification”); we further had, at the time of performing the experiment, included a further in vitro transcript spike-in selectively in some of the wells. In our protocol, several wells (some with, others without the additional spike-in) are pooled before the PCR step. If residual primer remained until PCR stage, UMI switches would also result in cell barcode switches; however, we observe such events only at very low frequency (new Figure E2e) and conclude that UMI switches do not affect the BATSeq data quality.

*A now standard metric for single cell techniques is to **correlate the 'in silico' average of single cells with a population experimen**t. Did the authors generate population data (i.e. from 48, or even 10,000 pooled mESC). It would be valuable to see the correlation of averaged single cell and population measurements, both for gene expression, and for 3' UTR usage.*

Even though we had not done so originally, we now added population data from two replicates of 48 ESC-2i cells each (new Figure E2f) to facilitate comparison with other methods. Correlations observed at the level of gene expression are in line with what has been reported for other methods (see e.g. Shalek et al., 2013). At the level of isoforms, correlations drop; however, this is mostly because many isoforms exist at very low abundance, as we and others have observed previously on bulk data (see Pelechano et al., 2013, Derti et al., 2011) and we do not consider these plots to contain much additional information beyond our new Figure E2f. We nevertheless show them here for the reviewer’s appraisal:

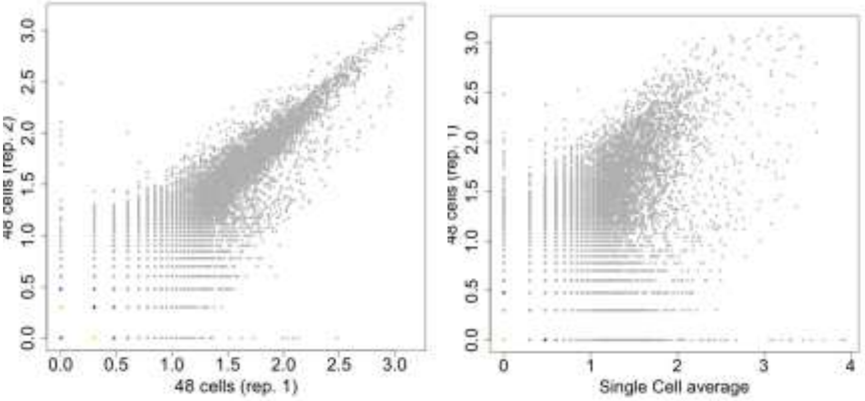

Pearson correlations are 0.78 and 0.61, respectively.

2. Cell identity can be inferred on based on 3' UTR usage alone.

*This is an interesting result, especially as this inference is performed only based on fractional UTR usage, and does not include gene expression. As an important control, the authors **should show that this result holds even when only considering genes that are expressed at similar levels** (i.e. average expression within 2 fold) between mESC and NSC. This is to control for the inherent fact (as the authors extensively describe) that noise in UTR estimation (both biological and technical) is highly dependent on expression abundance.*

We added the control suggested by the reviewer (new Figure E9c). Even when restricting our analysis to genes that differ no more than 2-fold in terms of expression level, cell types clearly cluster apart. Together with the simulation-based control implemented earlier (Figure E9b), we believe that we have convincingly shown that cell types can be distinguished based on their 3' UTR usage alone.

3. Variability in isoform preference across 'homogeneous' cells.

- *My understanding is that the BatBayes model only infers a global parameter for heterogeneity in 3' UTR usage, rather than inferring a gene by gene estimate of single cell variation. If this is correct, the authors should state this more clearly in the text.*

The BATBayes model shares information across genes to infer the variability in isoform preference for the "typical" gene, but also infers gene-wise estimates of isoform preference variability. However, we found only evidence for relatively minor gene-gene difference in isoform choice variability (Figure 4e, lower panel and Figure E7f+g). Larger isoform-specific single-cell sequencing datasets may in the future help to more clearly disentangle gene-wise differences in isoform choice variability. The BATBayes model can be applied for this type of analysis. We have highlighted that point in the main text. Also, we added a new figure 4b that depicts the BATBayes model as a directed acyclical graph to more clearly show what variables it infers.

It seems possible that a cell may choose to express long isoforms for some genes, and shorter versions of others. Another cell may make completely opposite isoform choices, but both cells would have the same average 3' UTR length. Would BatBayes accurate capture the heterogeneity in this scenario?

The BATBayes model is ignorant about 3’ UTR length, and we never infer a “mean UTR length” parameters. The data we applied to the model did not contain any information as to which isoform was long, or short. As Figure 6d shows, coordinate 3’ UTR lengthening from ESC to NSC is a strong trend, but there are exceptions, which were successfully identified by BATBayes2. Further, all the data created for the simulations (e.g. Figures 4d, E7 and E9) was created without specific length trends in mind, and heterogeneity was accurately captured.

I see that the authors do implement a frequentist control where heterogeneity/significance is calculated on the gene level. However, while there is a significant p-value, the results are not terribly convincing (i.e. not an obvious visual skew below the line in Fig. E7e). Do the authors' observe any functional enrichments for genes that are 'significantly heterogeneous' by this metric, or any correlations in the isoform choice for these genes across single cells?

We do not observe a functional enrichment on the level of RNA binding protein motifs, miRNA motifs, de novo motifs, or biological pathways. However, we are skeptical about such analysis for the following reasons: first, the number of genes that can be reliably looked at (at least two isoforms at noticeable expression in single cells) is relatively small (493 genes), impeding enrichment analyses as suggested by the reviewer. Second, our Bayesian modeling revealed that the data contains no evidence for the isoform preference variability to largely differ across genes (whether genes lie above or below the line in Fig E7e is, at the level of individual genes, probably mostly due to technical and sampling noise). While larger datasets may allow for a more detailed characterization, our findings can be explained by a mechanism that affects all genes, possibly acting co-transcriptionally (cf. Figure 8) or chromatin-mediated.

Reviewer #2:

The manuscript "Single cell polyadenylation site mapping reveals 3' isoform choice variability" by Velten et al., where an approach termed BATseq is employed to perform quantitative polyadenylation analysis in single mouse embryonic and neural stem cells.

Given the effort required to achieve BATseq libraries, I was **surprised that the authors only sequenced to a depth of ~40 million reads** (of which ~10% were used). Sequencing the samples to higher depth would likely have revealed considerably more poly-adenylation examples and complexity, and this additional coverage would surely have improved data quality, and the resulting analysis being more robust and increase the many different aspects of the manuscript substantially.

In line with what others have reported before (Jaitin et al., 2014; Pollen et al., 2014), we found that sequencing deeper into our libraries did not increase the number of molecules observed. This is because losses during reverse transcription and amplification limit complexity. Unlike previous work, we constructed our libraries on magnetic beads and were able to increase complexity by repeating the final PCR amplification step several times. These findings are reported in Figure E2a, but we have now highlighted them more clearly in the main text.

We estimate that we currently observe ca. 75% of the molecules present in the sequencing libraries. We use approximately 25% of sequencing reads (see Figure E2b).

A second broad recommendation would be that the authors, having established a technique and statistical framework for analyzing single-cell poly-adenylation events, should provide further evidence on how this approach provide novel biological insight. For example, what is the variation in the choice of isoforms between multiple cells of a single type, relative to what is the variation between multiple cells of different cell types? Is it possible to identify enriched sequence motifs associated with specific poly-adenylation trends **within** cell lines etc. ? Is there any correlation between the variation in 3UTR choice and variation in alternative splicing within a gene? Do specific classes of genes or developmental stages show greater variation in polyadenylation?

We thank the reviewer for these ideas. We have extended our analyses accordingly and attempt to communicate findings more clearly. To provide concise answers point by point:

What is the variation in the choice of isoforms between multiple cells of a single type, relative to what is the variation between multiple cells of different cell types?

We identified two types of variation, correlated and non-correlated variation. Correlated variation affects multiple genes and dominates between cells of different type, where we observe a global lengthening of 3' UTRs during developmental progression. Correlated variation of any kind (not restricted to coordinate 3' UTR lengthening) is not apparent between cells of the same type (see new Figure E9d).
By contrast, non-correlated variation is a type of variation that affects genes individually. Quantitatively, it is similar in magnitude in all cell types studied (Figure 4c).
While developmental regulation of poly-A site choice occurs in a coordinate fashion, noise in homogeneous populations apparently acts predominately *in cis*.

Do specific classes of developmental stages show greater variation in polyadenylation?

Non-correlated variation is similar in magnitude in all cell types studied (Figure 4c). It has recently been proposed that gene expression noise should be larger in the most pluripotent stem cells (MacArthur & Lemischka, 2013), however experimental evidence is not conclusive (Grün et al., 2014; Singer et al., 2014; Figure E5 of this work). It would therefore be relatively surprising if such effects were immediately evident at the level of polyadenylation site choice.

Is it possible to identify enriched sequence motifs associated with specific poly-adenylation trends **within** cell lines etc.? Do specific classes of genes show greater variation in polyadenylation?

We could imagine two trends to look for: First, are there genes whose isoform choice varies in a correlated manner within a cell population? – no such correlations are apparent within cells of the same type (see new Figure E9d), indicating that regulation occurs predominantly *in cis*.
Second, are there genes that are more variable than others? – an

obvious predictor of isoform usage variability is expression level (Figure 4e), but beyond that, we found relatively small differences across genes (Figure 4e, Figure E7f+g). We tested whether high-variable and low-variable genes differ in sequence motifs, but found no results, possibly due to the relatively small number of genes (493 genes) studied here. Based on the finding that active sites of transcription are dominated by single mRNA isoforms, we propose that isoform choice variability acts co-transcriptionally and affects all genes independently.

Is there any correlation between the variation in 3UTR choice and variation in alternative splicing within a gene?

The data we produced with the current version of BATSeq only allows us to make statements about 3' UTR choice. Modifications to the protocol that allow to investigate splicing in the same cell also are conceivable, but would require sequencing through the entire transcript, something that isn't yet possible at the throughput required here and lies beyond the scope of this study.

These are some examples of avenues that could be further investigated to provide insight to the mechanisms of polyadenylation.

Finally, I would like to commend the authors on a well-executed study. The manuscripts figures, text and presentation are all clear, well-written and accessible.

Minor Points:
1. Gene names are not italicized on several pages (eg. Page 6 Nanog, Dnmt3l. Lefty1, Stella, and Sca-1)
2. I found interpreting the paragraph at top of page 7 a little bit confusing. My understanding is that the ESC-2i population display several genes with highly variable polyadenylation. However, the number of identified variable genes were smaller in ESC-2i than in ESC-FCS. Yet the authors confirm that ES maintained in 2i medium do not constitute a completely homogenous population. However, this seems a contradictory, with ESC-2i being more homogenous than ESC-FCS. Could this please be clarified?

The indicated paragraph looks at gene expression levels, not polyadenylation variability. We found that even in 2i medium, gene expression for some genes is more variable than expected by technical noise. We agree with the reviewer that the statement about "2i cells being a completely homogeneous population" was misleading and we have changed it accordingly.

2nd Editorial Decision 15 February 2015

Thank you again for submitting your work to Molecular Systems Biology and apologies for a slow process with this new submission, which was mainly due to the Christmas break and the late arrival of both reviewers' reports. We have now finally heard back from the two referees whom we asked to evaluate your new submission. As you will see from the reports below, substantial concerns are raised on your work, which, I am afraid to say, preclude its publication in Molecular Systems Biology.

Thus, reviewer #1 is not convinced that the main conclusions are well supported and raises serious issues with regard to the interpretation of the data and the robustness of the conclusions. While the critiques about the interpretation of the RNA FISH are of perhaps lesser importance, given that a full mechanistic elucidation would be beyond the scope of this study, the issues related to the single-cell RNA-seq data are serious. While there is interest for the topic of the study, I am afraid that reviewer #1 provided the clear recommendation that the study should not be published in Molecular Systems Biology.

Under these circumstances, I see no other choice than to return the manuscript with the message that we cannot offer to publish it. I am very sorry not to be able to bring better news on this occasion. In any case, thank you for the opportunity to examine your work and I hope that the points raised in the reports will prove useful to you.

Reviewer #1:

The authors have added RNA FISH experiments as well as additional control experiments and analyses in this revised manuscript. Again, I feel that the strength of the manuscript is the statistical method devised to partition the observed variation into technical noise, random partitioning, and biological variation. I also apologize for not having previously observed the exonuclease cleanup step described in the supplementary methods.

However, significant concerns remain about the quality of the RNA-seq data presented in this manuscript, in part because of some of the new Figures that were generated in the reviewer response. Additionally, the authors draw significant new biological conclusions (in particular, that isoform choice is determined co-transcriptionally)- these conclusions have significant implications for the molecular mechanisms governing post-transcriptional regulation, but are not well-supported by these data.

In particular, Reviewer Response Figure 1b - which shows the correlation between the 'in silico average' of single cell measurements, and a population control raises a red flag. This Figure is a basic sanity check - in order for the data to be believed, the average measurement across single cells must be well correlated with a population measurement - both for gene expression and 3' UTR usage. This type of Figure is critical for the full measurement and should be in the main text, as it has been generated for each of the single cell RNA-seq papers the authors cite.

The Response Figure 1b here shows that only ~36% of the variance in the average isoform expression (as measured by the population dataset) - can be explained by single cell measurements. These numbers are far below previously published studies with ~ 48 cells, and challenges a central assumption of the authors' statistical model - that the only source of technical noise in the data is unbiased capture efficiency which equally affects each transcript. Though the authors claim that the weak correlations are due primarily to low-abundance isoforms, it is clear from the Figure that there is extensive disagreement for highly expressed isoforms as well.

Is it possible that another source of this low correlation is that there is also extensive noise in the 'population' experiment - which was done from only 48 cells? What if the authors generated a population experiment from a pool of thousands of cells, where the measurements could be assumed to be highly accurate? If this significantly increased the correlation for this Figure, that would alleviate this concern.

Either way, extensive noise in the data will obscure true correlative patterns between isoform choice across single cells. Thus, the authors cannot conclude that the primary drivers of isoform choice are in cis- indeed they suggest later that larger and more accurate datasets in the future are likely to reveal further regulatory insights (which could include potential trans associations). Similarly, the authors' claim that isoform choice occurs co-transcriptionally suggests a previously undiscovered coupling between 3' UTR selection and transcriptional activation. While this is interesting, this conclusion draws heavily upon RNA FISH data for 1 gene, the ability to link FISH fluorescence to raw molecule count (a technically challenging and unvalidated problem), and the assumption that there are little to no trans effects which govern 3' UTR usage. Thus, I do not believe that these conclusions are supported by the current data, and should be reworded/removed.

Reviewer #2:

Dear Editor,

Please find below my review of 'Single cell polyadenylation site mapping reveals 3' isoform variability".

The authors have a much-improved manuscript, and I would recommend its publication. The additional smFISH analysis adds additional depth to the study, and the authors have done a commendable job in preparing the manuscript and I quite enjoyed and congratulate the authors on the study.

I have made some suggestion however, that may aid in the readability of the manuscripts and its appeal to a broader biology community whom may not share the same statistical grounding of the authors.

1. Throughout the manuscript the authors refer to the different isoforms that result from variation in polyadenylation. However, there are a host of post-transcriptional processes that generate multiple isoforms, most notably splicing. While it would be cumbersome to refer to polyadenylated isoforms throughout the manuscript, I would suggest that this clarification is made in the abstract and the introduction of the manuscript. For example, in the abstract:

"While heterogeneity of gene expression has been extensively studied, little attention has been paid to mRNA isoform choice"

In this case, attention has been paid to isoforms due to splicing, but not due to polyadenylation (that is one of the strengths of this study).

Also polyadenylation site regulation should not be used interchangeably with post-transcriptional regulation, a term which also encompasses a much broader suite of processes.

2. I would be cautious about omitting false-positive polyadenylation sites outside the 12nt window which is based on analysis of ERCC poly-A sites. While this may capture the variation due to the sequencing process, the ERCC Spike-Ins have a encoded polyA tail, and do not undergo the biological process of polyadenylation, and therefore do not capture variation that may be associated with the biological process.

3. Could I suggest transferring the section titled "Strong isoform preferences in active sites of transcription" directly after "Isoform choice variability is also evident from smFISH". While the first examples the use of smFISH and it support for the model, the second section goes further in interpreting the smFISH analysis.

3. The final p[paragraph of the section entitled 'Coordinate changes in 3'UTR length dominate isoform preference in mixed populations'.

4. I have some difficulty in understanding the authors use of cis-acting in the manuscript and letter response. I interpret (possibly incorrectly) that the polyA site choice is largely noisy and independent (for lack of a better word) between each gene within the ESC-2i cell sample, and

therefore likely mediated by local cis-acting factors that are independent with other genes. Furthermore, no broader correlated trends, that would be enacted by global trans-factors, can be identified. Therefore, within the same cell type, the noisy polyadenylation site usage is largely defined by cis-factors.

However, in the context of this conclusion, I would have considered all polyadenylation site usage for all genes (whether correlated or not) to be mediated by both cis-acting mechanisms (co-transcriptional factors or local sequence elements) and trans-acting factors (the receptive protein environment which acts on the local cis-factors). Is. cis-factors at the polyadenylation site are the mechanism by which a trans-factors act.

While I do not disagree with the authors use of cis- and trans-, I include this example to indicate how using this quite loaded terminology can impact on a clear understanding of the authors conclusions, and it would be my suggestion that authors avoid using the cis- and trans- terminology altogether.

Minor Point

5. A schematic diagram of the isoforms and the probe sites in Figure 7E would aid interpretation by the reader.

Re-submission 26 March 2015

(see next page)

Molecular Systems Biology
Meyerhofstrasse 1
69117 Heidelberg

Lars M. Steinmetz, Ph.D.

Professor of Genetics
Stanford University School of Medicine
Department of Genetics
Stanford, CA 94305
USA

Co-Director
Stanford Genome Technology Center
3165 Porter Drive
Palo Alto, CA 94304
USA

Associate Head and Senior Scientist
European Molecular Biology Laboratory
Genome Biology Unit
Meyerhofstrasse 1
69117 Heidelberg
Germany

larsms@embl.de
larsms@stanford.edu
Tel. +49 6221 387389
Assistant, Sabine Blum
(sabine.blum@embl.de)

http://steinmetzlab.embl.de
http://steinmetzlab.stanford.edu

19.3.2015

Appeal regarding the Manuscript "Single Cell Polyadenylation Site Mapping"

Dear Thomas,

following concerns of reviewer #1 about the methodology presented in our manuscript *Single cell polyadenylation site mapping reveals 3′ isoform choice variability*, you rejected our work.

The focus of the reviewer's criticism was on the correlation between the sum of 48 single-cell transcriptomes ("in-silico pool") with the transcriptome of 48 cells ("bulk experiment"). Indeed, the correlations we observed (0.76 in the case of genes, 0.61 in the case of isoforms) were relatively low. We were not critical enough against these values, as similar (gene-level) correlation values (of 0.8) had been published before, in landmark papers of the field (e.g. Shalek et al., 2013).

However, following the reviewer's criticism, we noticed that

a) the control experiment we had included in the manuscript

European Molecular
Biology Laboratory

Laboratoire Européen
de Biologie Moléculaire

Europäisches Laboratorium
für Molekularbiologie

was problematic for two reasons: The bulk experiment was performed on a different day from the single-cell experiment, and a different cell lysis strategy had been used for the bulk experiment (as recommended by Sasagawa et al. 2013, the method that BATSeq is based on).

b) the correlations, if simulated from our model's assumptions of technical noise, should be much higher (~0.88 for genes and 0.78 for isoforms) than what we originally observed, indicative of problems with the control experiment.

We therefore repeated both the bulk experiment and 48 single cell experiments on the same day, using an identical lysis strategy. **This lead to much-improved correlations of 0.86 and 0.75 for genes and isoforms, respectively (new Figure 2d)**. Importantly, these values are very close to the values obtained from simulations, **verifying that the assumptions that were made during modelling capture all technical variance of BATSeq**.

We would kindly ask you to re-consider your decision to reject the manuscript based on the new data.

Below, we address all other (relatively small) points raised by the reviewers.

Do not hesitate to contact me should you have further questions.

Sincerely,

Lars Steinmetz, Ph.D.
Professor of Genetics, Stanford University

Co-Director, Stanford Genome Technology Center
Associate Head and Senior Scientist, EMBL

Reviewer #1:

The authors have added RNA FISH experiments as well as additional control experiments and analyses in this revised manuscript. Again, I feel that the strength of the manuscript is the statistical method devised to partition the observed variation into technical noise, random partitioning, and biological variation. I also apologize for not having previously observed the exonuclease cleanup step described in the supplementary methods.

However, significant concerns remain about the quality of the RNA-seq data presented in this manuscript, in part because of some of the new Figures that were generated in the reviewer response. Additionally, the authors draw significant new biological conclusions (in particular, that isoform choice is determined co-transcriptionally)- these conclusions have significant implications for the molecular mechanisms governing post-transcriptional regulation, but are not well-supported by these data.

In particular, Reviewer Response Figure 1b - which shows the correlation between the 'in silico average' of single cell measurements, and a population control raises a red flag. This Figure is a basic sanity check - in order for the data to be believed, the average measurement across single cells must be well correlated with a population measurement - both for gene expression and 3' UTR usage. This type of Figure is critical for the full measurement and should be in the main text, as it has been generated for each of the single cell RNA-seq papers the authors cite.

The Response Figure 1b here shows that only ~36% of the variance in the average isoform expression (as measured by the population dataset) - can be explained by single cell measurements. These numbers are far below previously published studies with ~ 48 cells, and challenges a central assumption of the authors' statistical model - that the only source of technical noise in the data is unbiased capture efficiency which equally affects each transcript. Though the authors claim that the weak correlations are due primarily to low-abundance isoforms, it is clear from the Figure that there is extensive disagreement for highly expressed isoforms as well.

As detailed above, there were several technical shortcomings of that control and we repeated the experiment. The new correlation observed for isoforms is 0.75 (0.86 at the gene level). If one simulates the data from our model's assumptions as detailed in the legend of Figure 2d, one obtains a correlation of 0.78 (0.88 at the gene level); the very minor discrepancy between these values can be explained by residual biological variability across two samples of 48 cells.

Importantly, the new correlation is now somewhat higher that values reported for other methods; also, the comparison to simulated data shows that our model captures all aspects of technical variability.

This control is now shown in Figure 2d and discussed in the main text.

Is it possible that another source of this low correlation is that there is also extensive noise in the 'population' experiment - which was done from only 48 cells? What if the authors generated a population experiment from a pool of thousands of cells, where the measurements could be assumed to be highly accurate? If this significantly increased the correlation for this Figure, that would alleviate this concern.

We refrained from repeating the population experiment for a higher number of cells because

a) The new correlations reported above provide a significantly increase correlation already, with very little residual biological variance
b) Pooling a large number of cells in the very small volume of BATSeq lysis buffer (0.6µL) may be problematic, also because the buffer will become considerably diluted by liquid from the FACS droplets. Using a different lysis strategy (e.g. Qiagen buffer RLT followed by Ampure cleanup, as we had done before) is likely to create systematic differences between the two experiments.
c) Sequencing a higher number of cells for the bulk experiment may actually decrease correlations, because many lowly expressed genes will be captured only in the 1000s-cell sample, but not in the 48-cell sample (thus adding considerable Poisson noise to the lower part of the scatter plot along the 1000s-cell axis; see e.g. Shalek et al. 2013, Figure 1c).

Either way, extensive noise in the data will obscure true correlative patterns between isoform choice across single cells. Thus, the authors cannot conclude that the primary drivers of isoform choice are in cis- indeed they suggest later that larger and more accurate datasets in the future are likely to reveal further regulatory insights (which could include potential trans associations). Similarly, the authors' claim that isoform choice occurs co-transcriptionally suggests a previously undiscovered coupling between 3' UTR selection and transcriptional activation. While this is interesting, this conclusion draws heavily upon RNA FISH data for 1 gene, the ability to link FISH fluorescence to raw molecule count (a technically challenging and unvalidated problem), and the assumption that there are little to no trans effects which govern 3' UTR usage. Thus, I do not believe that these conclusions are supported by the current data, and should be reworded/removed.

We moved the corresponding Figure (formerly Figure 7) to the supplement, and briefly mention that observation as initial data that

points towards, but does not proof, the proposed mechanism. The discussion is also modified accordingly.

Reviewer #2:

1. Throughout the manuscript the authors refer to the different isoforms that result from variation in polyadenylation. However, there are a host of post-transcriptional processes that generate multiple isoforms, most notably splicing. While it would be cumbersome to refer to polyadenylated isoforms throughout the manuscript, I would suggest that this clarification is made in the abstract and the introduction of the manuscript. For example, in the abstract:

"While heterogeneity of gene expression has been extensively studied, little attention has been paid to mRNA isoform choice"

In this case, attention has been paid to isoforms due to splicing, but not due to polyadenylation (that is one of the strengths of this study).

Also polyadenylation site regulation should not be used interchangeably with post-transcriptional regulation, a term which also encompasses a much broader suite of processes.

> We now stress more clearly that the study is concerned with polyadenylation isoforms in the abstract and the final paragraph of the introduction; we believe that the first part of the introduction very clearly explains that the study deals with alternative polyadenylation.

2. I would be cautious about omitting false-positive polyadenylation sites outside the 12nt window which is based on analysis of ERCC poly-A sites. While this may capture the variation due to the sequencing process, the ERCC Spike-Ins have a encoded polyA tail, and do not undergo the biological process of polyadenylation, and therefore do not capture variation that may be associated with the biological process.

> The 12 bp window does not serve to omit sites. It is only used to merge sites that are in immediate vicinity; while it is true that biological variation may also create 3' isoforms that differ in length by single nucleotides, the ERCC control shows that we are technically unable to reliable resolve such variation. Therefore, we collapse PA sites within a 12 bp window. Reads mapping outside the 12bp window define new polyadenylation sites.

3. Could I suggest transferring the section titled "Strong isoform preferences in active sites of transcription" directly after "Isoform choice variability is also evident from smFISH". While the first examples the use of smFISH and it support for the model, the second section goes further in interpreting the smFISH analysis.

> Following the criticism of reviewer 1, we much shortened that section and include it as a single paragraph in *Isoform choice variability is also evident from smFISH*. The corresponding figure was moved to supplement. This is to make clear that while interesting, this process warrants further investigation. We have also reworded our discussion accordingly.

3. The final p[paragraph of the section entitled 'Coordinate changes in 3'UTR length dominate isoform preference in mixed populations'.

> Was there a copy-paste error in the reviewer's report? It is unclear to us what the reviewer suggests.

4. I have some difficulty in understanding the authors use of cis-acting in the manuscript and letter response. I interpret (possibly incorrectly) that the polyA site choice is largely noisy and independent (for lack of a better word) between each gene within the ESC-2i cell sample, and therefore likely mediated by local cis-acting factors that are independent with other genes. Furthermore, no broader correlated trends, that would be enacted by global trans-factors, can be identified. Therefore, within the same cell type, the noisy polyadenylation site usage is largely

defined by cis-factors.

However, in the context of this conclusion, I would have considered all polyadenylation site usage for all genes (whether correlated or not) to be mediated by both cis-acting mechanisms (co-transcriptional factors or local sequence elements) and trans-acting factors (the receptive protein environment which acts on the local cis-factors). Is. cis-factors at the polyadenylation site are the mechanism by which a trans-factors act.

While I do not disagree with the authors use of cis- and trans-, I include this example to indicate how using this quite loaded terminology can impact on a clear understanding of the authors conclusions, and it would be my suggestion that authors avoid using the cis- and trans-terminology altogether.

We have removed the cis/trans terminology altogether.

Minor Point

5. A schematic diagram of the isoforms and the probe sites in Figure 7E would aid interpretation by the reader.

We have added the schematic suggested by the reviewer.

3rd Editorial Decision 22 April 2015

Thank you again for submitting your amended work to Molecular Systems Biology. We have now finally heard back from one of our Board members with whom we consulted on the added data showing a much better correlation between the averaged single cell data and the population data. Our advisor agreed that the correlation shown was good and indicated the data is of good quality. I am pleased to inform you that we will be able to accept your manuscript for publication and I would thus ask you to submit the final version of the paper.

1st Revision - authors' response 24 April 2015

Following the update of the MSB Author Guidelines, we reformatted our manuscript to include only four Expanded View figures and two Expanded View tables. All further supplementary figures, tables, and text were moved to an Appendix. Other than that, there were only very minor edits to the manuscript.
